# Supplementary material for: Relative Effect of Extracorporeal Shockwave Therapy Alone or in Combination with Noninjective Treatments on Pain and Physical Function in Knee Osteoarthritis: A Network Meta-Analysis of Randomized Controlled Trials
Source: Biomedicines. 2022 Jan 28;10(2):306. doi: 10.3390/biomedicines10020306 (PMC8869515; doi:10.3390/biomedicines10020306)

**Figure S1**

### Overall follow up duration

| Treatment Reference to UC | Direct Comparisons | SMD | 95% CI | P−score |
|---|---|---|---|---|
| LI−RaSW | 2 | −0.04 | [−0.79; 0.71] | 0.07 |
| LI−RaSW + CPT | 2 | −0.97 | [−2.39; 0.46] | 0.31 |
| MI−RaSW + CPT | 6 | −1.18 | [−1.94; −0.41] | 0.35 |
| HI−FoSW | 5 | −1.21 | [−2.15; −0.26] | 0.37 |
| MI−RaSW (Acupoint) | 2 | −1.31 | [−2.39; −0.23] | 0.40 |
| LI−FoSW | 1 | −1.29 | [−2.91; 0.34] | 0.41 |
| LI−RaSW + TCM | 1 | −1.38 | [−2.69; −0.07] | 0.44 |
| LI−FoSW + TCM | 1 | −1.37 | [−3.46; 0.72] | 0.44 |
| HI−FoSW + CPT | 1 | −1.39 | [−3.47; 0.69] | 0.45 |
| MI−RaSW (Acupoint) + CPT | 0 | −1.50 | [−4.64; 1.63] | 0.47 |
| MI−RaSW | 5 | −1.61 | [−2.36; −0.86] | 0.52 |
| HI−FoSW + TCM | 1 | −1.67 | [−3.78; 0.45] | 0.52 |
| HI−RaSW | 12 | −1.64 | [−2.19; −1.10] | 0.53 |
| HI−RaSW + CPT | 4 | −1.74 | [−2.70; −0.78] | 0.57 |
| MI−FoSW + TCM | 1 | −1.93 | [−3.83; −0.04] | 0.60 |
| MI−RaSW + TCM | 1 | −2.09 | [−4.20; 0.02] | 0.64 |
| MI−FoSW | 4 | −1.95 | [−2.95; −0.95] | 0.64 |
| LI−FoSW + CPT | 1 | −2.23 | [−4.49; 0.04] | 0.66 |
| HI−RaSW + TCM | 3 | −2.29 | [−3.29; −1.29] | 0.74 |
| MI−RaSW (Acupoint) + TCM | 0 | −3.13 | [−5.48; −0.78] | 0.84 |
| MI−FoSW + CPT | 0 | −4.51 | [−6.85; −2.18] | 0.97 |

Effects on pain reduction Random Effects Model

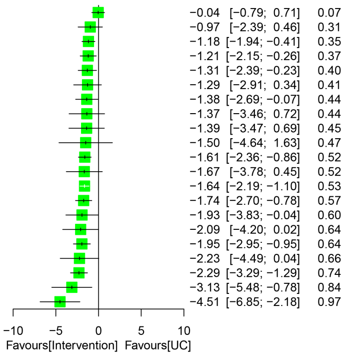


### Follow up ≥ 6 months

| Treatment Reference to UC | Direct Comparisons | SMD | 95% CI | P−score |
|---|---|---|---|---|
| LI−RaSW | 0 | 1.16 | [−0.60; 2.92] | 0.10 |
| HI−RaSW + CPT | 0 | 1.13 | [−1.34; 3.59] | 0.14 |
| MI−RaSW (Acupoint) | 1 | −0.10 | [−1.81; 1.61] | 0.37 |
| HI−RaSW | 2 | −0.76 | [−1.78; 0.26] | 0.57 |
| HI−RaSW + TCM | 2 | −1.15 | [−2.29; −0.01] | 0.68 |
| HI−FoSW + CPT | 1 | −1.25 | [−2.97; 0.48] | 0.69 |
| HI−FoSW + TCM | 1 | −1.25 | [−3.01; 0.50] | 0.69 |
| MI−RaSW | 2 | −2.40 | [−3.50; −1.30] | 0.96 |

Effects on pain reduction Random Effects Model

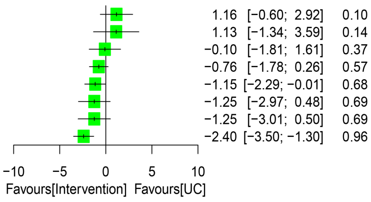


### Follow up ≥3 months, < 6 months

| Treatment Reference to UC | Direct Comparisons | SMD | 95% CI | P−score |
|---|---|---|---|---|
| LI−RaSW | 0 | 0.18 | [−1.47; 1.84] | 0.14 |
| HI−RaSW + CPT | 1 | 0.12 | [−1.33; 1.57] | 0.15 |
| MI−RaSW (Acupoint) | 1 | −0.55 | [−1.97; 0.87] | 0.35 |
| HI−RaSW | 7 | −1.04 | [−1.65; −0.42] | 0.50 |
| MI−RaSW | 2 | −1.26 | [−2.44; −0.08] | 0.58 |
| LI−RaSW + CPT | 1 | −1.32 | [−3.06; 0.43] | 0.59 |
| MI−RaSW + CPT | 0 | −1.31 | [−3.34; 0.72] | 0.61 |
| HI−RaSW + TCM | 3 | −1.40 | [−2.29; −0.52] | 0.64 |
| HI−FoSW + TCM | 1 | −1.67 | [−3.36; 0.03] | 0.69 |
| LI−FoSW | 0 | −1.88 | [−4.26; 0.51] | 0.71 |
| MI−FoSW | 1 | −2.43 | [−4.13; −0.73] | 0.88 |

Effects on pain reduction Random Effects Model

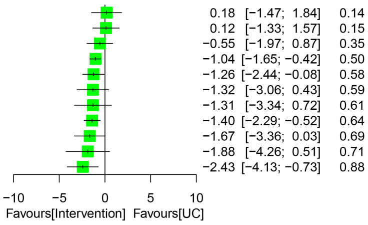


### Follow up ≥1 months, < 3 months

| Treatment Reference to UC | Direct Comparisons | SMD | 95% CI | P−score |
|---|---|---|---|---|
| LI−RaSW | 2 | −0.04 | [−0.83; 0.76] | 0.09 |
| HI−FoSW + TCM | 1 | −0.12 | [−2.06; 1.82] | 0.17 |
| LI−RaSW + CPT | 2 | −0.96 | [−2.29; 0.37] | 0.35 |
| MI−RaSW + CPT | 6 | −1.16 | [−1.87; −0.44] | 0.39 |
| HI−FoSW | 5 | −1.20 | [−2.08; −0.33] | 0.42 |
| MI−RaSW (Acupoint) + CPT | 0 | −1.20 | [−4.31; 1.90] | 0.44 |
| MI−RaSW | 5 | −1.30 | [−2.02; −0.59] | 0.45 |
| MI−RaSW (Acupoint) | 0 | −1.32 | [−2.81; 0.17] | 0.46 |
| HI−RaSW | 11 | −1.36 | [−1.89; −0.84] | 0.47 |
| HI−FoSW + CPT | 1 | −1.39 | [−3.32; 0.53] | 0.49 |
| LI−FoSW | 1 | −1.43 | [−2.97; 0.12] | 0.50 |
| HI−RaSW + TCM | 3 | −1.78 | [−2.70; −0.85] | 0.64 |
| MI−FoSW + TCM | 1 | −1.91 | [−3.68; −0.13] | 0.65 |
| MI−FoSW | 3 | −1.89 | [−2.94; −0.84] | 0.67 |
| MI−RaSW + TCM | 1 | −2.09 | [−4.05; −0.14] | 0.69 |
| LI−FoSW + CPT | 1 | −2.23 | [−4.35; −0.11] | 0.71 |
| HI−RaSW + CPT | 4 | −2.11 | [−3.10; −1.12] | 0.74 |
| LI−RaSW + TCM | 0 | −2.44 | [−4.58; −0.30] | 0.76 |
| MI−RaSW (Acupoint) + TCM | 0 | −2.83 | [−5.27; −0.39] | 0.83 |

Effects on pain reduction Random Effects Model

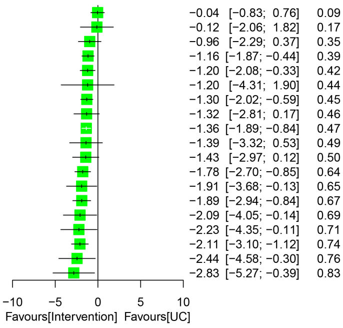


### Follow up < 1 month

| Treatment Reference to UC | Direct Comparisons | SMD | 95% CI | P−score |
|---|---|---|---|---|
| LI−RaSW | 0 | 0.29 | [−1.02; 1.61] | 0.11 |
| HI−FoSW + TCM | 1 | −0.10 | [−2.30; 2.10] | 0.24 |
| MI−RaSW + CPT | 3 | −0.39 | [−1.53; 0.75] | 0.27 |
| LI−RaSW + TCM | 1 | −0.70 | [−2.38; 0.98] | 0.37 |
| HI−RaSW + CPT | 1 | −0.88 | [−3.14; 1.39] | 0.42 |
| MI−RaSW | 2 | −1.00 | [−2.19; 0.18] | 0.45 |
| MI−RaSW (Acupoint) | 1 | −1.27 | [−2.70; 0.17] | 0.52 |
| LI−FoSW + TCM | 1 | −1.37 | [−3.56; 0.82] | 0.54 |
| MI−FoSW | 3 | −1.46 | [−2.74; −0.18] | 0.57 |
| MI−FoSW + TCM | 1 | −1.54 | [−3.56; 0.48] | 0.58 |
| HI−RaSW + TCM | 1 | −1.57 | [−3.52; 0.38] | 0.59 |
| LI−FoSW | 0 | −1.79 | [−4.33; 0.76] | 0.62 |
| HI−RaSW | 3 | −2.25 | [−3.36; −1.13] | 0.78 |
| MI−RaSW (Acupoint) + TCM | 0 | −3.09 | [−5.70; −0.47] | 0.85 |
| MI−FoSW + CPT | 0 | −4.02 | [−6.58; −1.47] | 0.95 |

Effects on pain reduction Random Effects Model

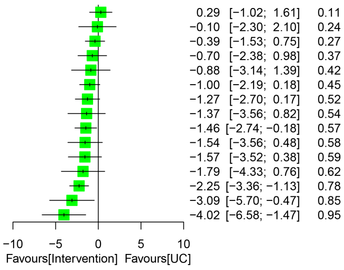

## Figure S2

### Overall follow up duration

| Treatment Reference to UC | Direct Comparisons | SMD | 95% CI | P−score |
|---|---|---|---|---|
| MI−RaSW (Acupoint) + TCM | 0 | 4.79 | [ 2.17; 7.41] | 0.97 |
| MI−RaSW (Acupoint) + CPT | 0 | 3.95 | [ 0.48; 7.41] | 0.87 |
| HI−RaSW + TCM | 3 | 2.50 | [ 1.41; 3.59] | 0.78 |
| MI−RaSW (Acupoint) | 1 | 2.46 | [ 1.21; 3.71] | 0.77 |
| MI−RaSW | 5 | 2.11 | [ 1.28; 2.93] | 0.70 |
| MI−FoSW + CPT | 0 | 2.29 | [−0.29; 4.87] | 0.67 |
| HI−FoSW + CPT | 1 | 1.97 | [−0.32; 4.26] | 0.61 |
| HI−RaSW + CPT | 4 | 1.71 | [ 0.67; 2.75] | 0.57 |
| MI−RaSW + CPT | 7 | 1.63 | [ 0.85; 2.42] | 0.54 |
| LI−RaSW + TCM | 0 | 1.63 | [−0.20; 3.47] | 0.54 |
| HI−RaSW | 13 | 1.54 | [ 0.96; 2.12] | 0.51 |
| MI−FoSW | 3 | 1.44 | [ 0.24; 2.64] | 0.48 |
| HI−FoSW | 5 | 1.41 | [ 0.38; 2.45] | 0.47 |
| MI−FoSW + TCM | 3 | 1.30 | [ 0.02; 2.58] | 0.44 |
| LI−FoSW + CPT | 1 | 0.93 | [−1.45; 3.32] | 0.32 |
| HI−FoSW + TCM | 1 | 0.75 | [−1.55; 3.05] | 0.32 |
| LI−RaSW + CPT | 2 | 0.84 | [−0.71; 2.39] | 0.32 |
| LI−RaSW | 2 | 0.51 | [−0.35; 1.37] | 0.21 |
| MI−RaSW + TCM | 2 | 0.13 | [−1.39; 1.65] | 0.15 |
| LI−FoSW | 1 | 0.05 | [−1.73; 1.84] | 0.15 |

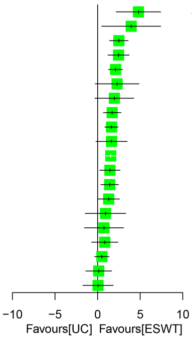


### Follow up ≥6 months

| Treatment Reference to UC | Direct Comparisons | SMD | 95% CI | P−score |
|---|---|---|---|---|
| MI−RaSW | 2 | 3.70 | [ 2.18; 5.21] | 0.93 |
| HI−RaSW + TCM | 2 | 2.83 | [ 1.26; 4.40] | 0.79 |
| HI−FoSW + CPT | 1 | 1.97 | [−0.38; 4.32] | 0.60 |
| HI−RaSW + CPT | 0 | 1.97 | [−1.37; 5.30] | 0.59 |
| LI−RaSW | 0 | 1.27 | [−1.09; 3.64] | 0.43 |
| HI−RaSW | 2 | 1.11 | [−0.28; 2.51] | 0.40 |
| MI−RaSW (Acupoint) | 1 | 1.01 | [−1.33; 3.34] | 0.39 |
| HI−FoSW + TCM | 1 | 0.42 | [−1.93; 2.78] | 0.26 |

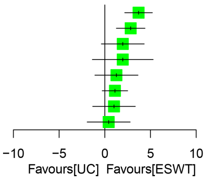


### Follow up ≥3 months, <6 months

| Treatment Reference to UC | Direct Comparisons | SMD | 95% CI | P−score |
|---|---|---|---|---|
| MI−RaSW + CPT | 0 | 5.75 | [ 2.57; 8.92] | 0.99 |
| MI−RaSW (Acupoint) | 0 | 2.68 | [ 0.25; 5.10] | 0.79 |
| HI−RaSW + TCM | 3 | 1.84 | [ 0.54; 3.15] | 0.66 |
| MI−FoSW | 1 | 1.88 | [−0.56; 4.32] | 0.64 |
| MI−RaSW | 2 | 1.43 | [−0.19; 3.06] | 0.54 |
| HI−RaSW | 6 | 1.30 | [ 0.32; 2.28] | 0.51 |
| LI−RaSW | 0 | 1.09 | [−1.47; 3.66] | 0.46 |
| HI−RaSW + CPT | 1 | 0.78 | [−1.38; 2.95] | 0.38 |
| LI−RaSW + CPT | 1 | 0.67 | [−1.81; 3.14] | 0.37 |
| LI−FoSW | 0 | 0.49 | [−2.97; 3.94] | 0.35 |
| HI−FoSW + TCM | 1 | −0.78 | [−3.21; 1.66] | 0.12 |

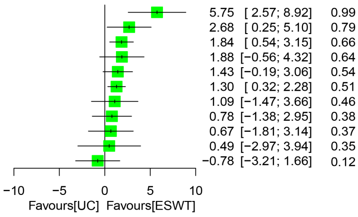


### Follow up ≥1 months, <3 months

| Treatment Reference to UC | Direct Comparisons | SMD | 95% CI | P−score |
|---|---|---|---|---|
| MI−RaSW (Acupoint) + TCM | 0 | 4.17 | [ 1.61; 6.73] | 0.96 |
| MI−RaSW (Acupoint) + CPT | 0 | 3.32 | [−0.06; 6.71] | 0.84 |
| MI−RaSW + CPT | 6 | 2.19 | [ 1.35; 3.02] | 0.80 |
| HI−RaSW + TCM | 3 | 1.99 | [ 0.92; 3.05] | 0.74 |
| MI−RaSW (Acupoint) | 1 | 1.84 | [ 0.61; 3.06] | 0.69 |
| HI−RaSW + CPT | 4 | 1.79 | [ 0.67; 2.91] | 0.68 |
| MI−RaSW | 5 | 1.56 | [ 0.75; 2.37] | 0.61 |
| HI−FoSW | 5 | 1.41 | [ 0.40; 2.43] | 0.56 |
| HI−RaSW | 13 | 1.27 | [ 0.70; 1.83] | 0.51 |
| LI−RaSW + TCM | 0 | 1.22 | [−1.21; 3.66] | 0.51 |
| HI−FoSW + CPT | 1 | 1.21 | [−1.02; 3.44] | 0.50 |
| MI−FoSW + TCM | 2 | 1.06 | [−0.44; 2.56] | 0.46 |
| LI−RaSW + CPT | 2 | 1.00 | [−0.52; 2.52] | 0.44 |
| LI−FoSW + CPT | 1 | 0.93 | [−1.40; 3.27] | 0.44 |
| MI−FoSW | 4 | 0.98 | [−0.07; 2.03] | 0.43 |
| MI−RaSW + TCM | 2 | 0.29 | [−1.20; 1.78] | 0.26 |
| LI−FoSW | 1 | −0.02 | [−1.74; 1.70] | 0.20 |
| LI−RaSW | 2 | −0.31 | [−1.22; 0.61] | 0.11 |
| HI−FoSW + TCM | 1 | −0.83 | [−3.08; 1.41] | 0.10 |

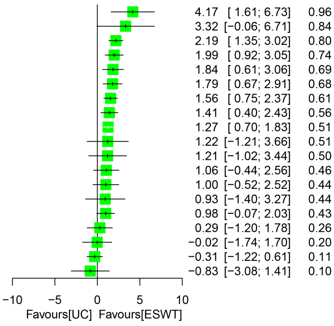


### Follow up <1 month

| Treatment Reference to UC | Direct Comparisons | SMD | 95% CI | P−score |
|---|---|---|---|---|
| MI−RaSW (Acupoint) + TCM | 0 | 2.78 | [ 0.54; 5.02] | 0.90 |
| MI−RaSW (Acupoint) | 0 | 2.00 | [ 0.60; 3.41] | 0.79 |
| MI−RaSW | 2 | 1.86 | [ 0.79; 2.94] | 0.77 |
| HI−RaSW + TCM | 1 | 1.54 | [−0.01; 3.09] | 0.65 |
| MI−FoSW + CPT | 0 | 1.58 | [−0.54; 3.71] | 0.65 |
| HI−RaSW | 3 | 1.39 | [ 0.49; 2.28] | 0.62 |
| MI−FoSW + TCM | 3 | 1.16 | [ 0.16; 2.16] | 0.54 |
| LI−RaSW + TCM | 0 | 0.90 | [−1.25; 3.05] | 0.45 |
| MI−RaSW + CPT | 3 | 0.85 | [−0.09; 1.78] | 0.42 |
| HI−FoSW + TCM | 1 | 0.75 | [−1.03; 2.52] | 0.40 |
| MI−FoSW | 2 | 0.73 | [−0.47; 1.93] | 0.38 |
| LI−FoSW | 0 | 0.64 | [−1.50; 2.78] | 0.37 |
| HI−RaSW + CPT | 1 | 0.19 | [−1.65; 2.04] | 0.25 |
| LI−RaSW | 0 | 0.16 | [−1.08; 1.40] | 0.19 |

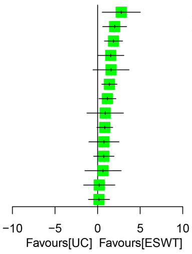

**Figure S3**

| Comparison | Number of Studies | Direct Evidence | I2 | SMD | 95%−CI |
|---|---|---|---|---|---|
| **HI−RaSW vs HI−RaSW + CPT** | | | | | |
| Direct estimate | 1 | 0.25 | | 1.16 | [ −0.96; 3.27] |
| Indirect estimate | | | | −0.25 | [ −1.46; 0.97] |
| Network estimate | | | | 0.10 | [ −0.95; 1.15] |
| **HI−RaSW vs HI−RaSW + TCM** | | | | | |
| Direct estimate | 3 | 0.69 | 0.75 | 1.71 | [ 0.51; 2.91] |
| Indirect estimate | | | | −1.72 | [ −3.52; 0.07] |
| Network estimate | | | | 0.65 | [ −0.35; 1.65] |
| **HI−RaSW vs LI−RaSW** | | | | | |
| Direct estimate | 2 | 0.32 | 0.79 | −2.89 | [ −4.41; −1.38] |
| Indirect estimate | | | | −0.99 | [ −2.03; 0.05] |
| Network estimate | | | | −1.60 | [ −2.46; −0.74] |
| **HI−RaSW vs MI−RaSW** | | | | | |
| Direct estimate | 2 | 0.34 | 0.98 | −0.48 | [ −1.95; 1.00] |
| Indirect estimate | | | | 0.19 | [ −0.86; 1.25] |
| Network estimate | | | | −0.03 | [ −0.89; 0.82] |
| **HI−RaSW vs UC** | | | | | |
| Direct estimate | 12 | 0.81 | 0.95 | −1.68 | [ −2.29; −1.07] |
| Indirect estimate | | | | −1.48 | [ −2.72; −0.24] |
| Network estimate | | | | −1.64 | [ −2.19; −1.10] |
| **HI−RaSW + CPT vs LI−RaSW** | | | | | |
| Direct estimate | 1 | 0.28 | | −0.03 | [ −2.11; 2.05] |
| Indirect estimate | | | | −2.37 | [ −3.68; −1.05] |
| Network estimate | | | | −1.70 | [ −2.81; −0.59] |
| **HI−RaSW + CPT vs UC** | | | | | |
| Direct estimate | 4 | 0.75 | 0.96 | −2.20 | [ −3.30; −1.09] |
| Indirect estimate | | | | −0.36 | [ −2.28; 1.56] |
| Network estimate | | | | −1.74 | [ −2.70; −0.78] |
| **HI−RaSW + TCM vs UC** | | | | | |
| Direct estimate | 3 | 0.70 | 0.93 | −1.28 | [ −2.47; −0.09] |
| Indirect estimate | | | | −4.64 | [ −6.46; −2.82] |
| Network estimate | | | | −2.29 | [ −3.29; −1.29] |
| **LI−FoSW vs MI−FoSW** | | | | | |
| Direct estimate | 1 | 0.59 | | 0.55 | [ −1.54; 2.65] |
| Indirect estimate | | | | 0.82 | [ −1.68; 3.32] |
| Network estimate | | | | 0.66 | [ −0.94; 2.27] |
| **LI−FoSW vs UC** | | | | | |
| Direct estimate | 1 | 0.52 | | −1.16 | [ −3.42; 1.11] |
| Indirect estimate | | | | −1.43 | [ −3.77; 0.92] |
| Network estimate | | | | −1.29 | [ −2.91; 0.34] |
| **LI−RaSW vs LI−RaSW + TCM** | | | | | |
| Direct estimate | 2 | 0.69 | 0.95 | 1.42 | [ −0.07; 2.91] |
| Indirect estimate | | | | 1.14 | [ −1.09; 3.37] |
| Network estimate | | | | 1.34 | [ 0.10; 2.58] |
| **LI−RaSW vs MI−RaSW** | | | | | |
| Direct estimate | 2 | 0.40 | 0.90 | 2.43 | [ 0.94; 3.93] |
| Indirect estimate | | | | 1.00 | [ −0.22; 2.21] |
| Network estimate | | | | 1.57 | [ 0.62; 2.51] |
| **LI−RaSW vs MI−RaSW (Acupoint)** | | | | | |
| Direct estimate | 1 | 0.32 | | 1.40 | [ −0.69; 3.49] |
| Indirect estimate | | | | 1.20 | [ −0.22; 2.63] |
| Network estimate | | | | 1.27 | [ 0.09; 2.45] |
| **LI−RaSW vs MI−RaSW + CPT** | | | | | |
| Direct estimate | 2 | 0.39 | 0.81 | 1.51 | [ 0.03; 2.99] |
| Indirect estimate | | | | 0.90 | [ −0.30; 2.09] |
| Network estimate | | | | 1.14 | [ 0.21; 2.07] |
| **LI−RaSW vs UC** | | | | | |
| Direct estimate | 2 | 0.25 | 0.95 | −1.09 | [ −2.57; 0.40] |
| Indirect estimate | | | | 0.32 | [ −0.55; 1.19] |
| Network estimate | | | | −0.04 | [ −0.79; 0.71] |
| **LI−RaSW + CPT vs MI−RaSW + CPT** | | | | | |
| Direct estimate | 1 | 0.48 | | 1.59 | [ −0.59; 3.77] |
| Indirect estimate | | | | −1.07 | [ −3.17; 1.03] |
| Network estimate | | | | 0.21 | [ −1.30; 1.73] |
| **LI−RaSW + CPT vs UC** | | | | | |
| Direct estimate | 2 | 0.87 | 0.00 | −1.53 | [ −3.06; 0.01] |
| Indirect estimate | | | | 2.71 | [ −1.22; 6.63] |
| Network estimate | | | | −0.97 | [ −2.39; 0.46] |
| **LI−RaSW + TCM vs UC** | | | | | |
| Direct estimate | 1 | 0.39 | | −1.21 | [ −3.30; 0.88] |
| Indirect estimate | | | | −1.49 | [ −3.17; 0.20] |
| Network estimate | | | | −1.38 | [ −2.69; −0.07] |
| **MI−FoSW vs MI−FoSW + TCM** | | | | | |
| Direct estimate | 1 | 0.81 | | 0.91 | [ −1.20; 3.02] |
| Indirect estimate | | | | −3.89 | [ −8.21; 0.44] |
| Network estimate | | | | −0.01 | [ −1.91; 1.88] |
| **MI−FoSW vs UC** | | | | | |
| Direct estimate | 4 | 0.89 | 0.96 | −1.98 | [ −3.03; −0.92] |
| Indirect estimate | | | | −1.71 | [ −4.79; 1.38] |
| Network estimate | | | | −1.95 | [ −2.95; −0.95] |
| **MI−FoSW + TCM vs UC** | | | | | |
| Direct estimate | 1 | 0.81 | | −1.01 | [ −3.12; 1.10] |
| Indirect estimate | | | | −5.80 | [−10.12; −1.48] |
| Network estimate | | | | −1.93 | [ −3.83; −0.04] |
| **MI−RaSW vs MI−RaSW (Acupoint)** | | | | | |
| Direct estimate | 1 | 0.32 | | 0.36 | [ −1.73; 2.44] |
| Indirect estimate | | | | −0.61 | [ −2.04; 0.82] |
| Network estimate | | | | −0.30 | [ −1.48; 0.88] |
| **MI−RaSW vs UC** | | | | | |
| Direct estimate | 5 | 0.62 | 0.95 | −1.64 | [ −2.60; −0.69] |
| Indirect estimate | | | | −1.55 | [ −2.77; −0.34] |
| Network estimate | | | | −1.61 | [ −2.36; −0.86] |
| **MI−RaSW (Acupoint) vs UC** | | | | | |
| Direct estimate | 2 | 0.54 | 0.87 | −0.92 | [ −2.38; 0.55] |
| Indirect estimate | | | | −1.77 | [ −3.37; −0.18] |
| Network estimate | | | | −1.31 | [ −2.39; −0.23] |
| **MI−RaSW + CPT vs UC** | | | | | |
| Direct estimate | 6 | 0.78 | 0.83 | −1.01 | [ −1.88; −0.15] |
| Indirect estimate | | | | −1.77 | [ −3.40; −0.14] |
| Network estimate | | | | −1.18 | [ −1.94; −0.41] |

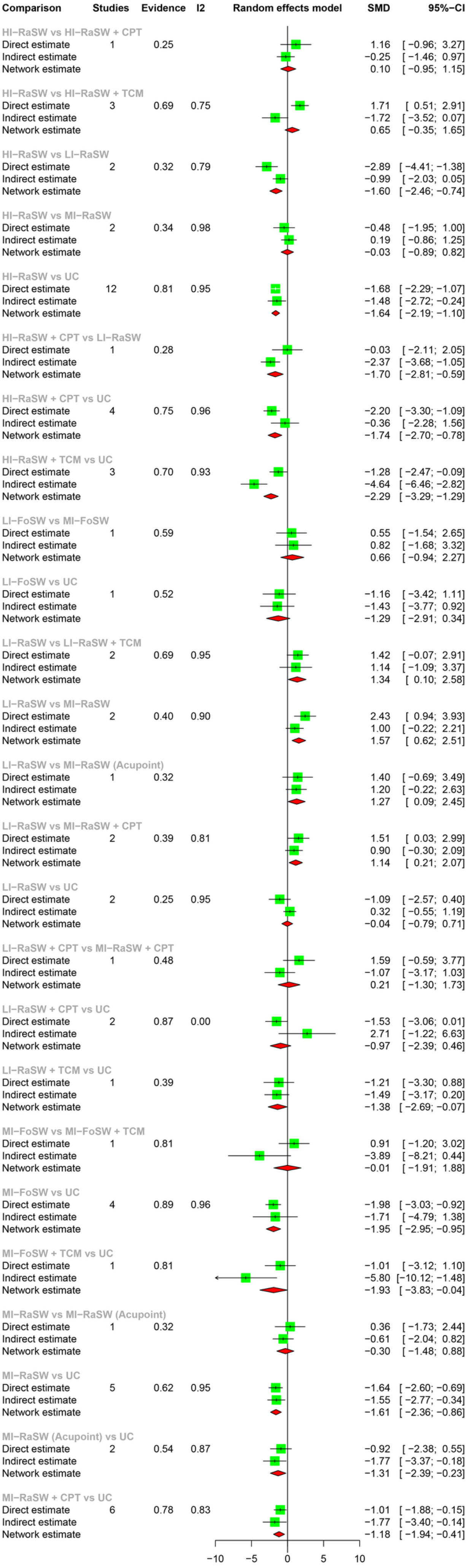

**Figure S4**

| Comparison | Number of Studies | Direct Evidence | I2 | SMD | 95%−CI |
|---|---|---|---|---|---|
| **HI−RaSW vs HI−RaSW + CPT** | | | | | |
| Direct estimate | 1 | 0.23 | | −3.32 | [ −5.69; −0.96] |
| Indirect estimate | | | | 0.78 | [ −0.52; 2.08] |
| Network estimate | | | | −0.17 | [ −1.31; 0.97] |
| **HI−RaSW vs HI−RaSW + TCM** | | | | | |
| Direct estimate | 3 | 0.69 | 0.94 | −1.44 | [ −2.75; −0.12] |
| Indirect estimate | | | | 0.10 | [ −1.86; 2.06] |
| Network estimate | | | | −0.96 | [ −2.05; 0.13] |
| **HI−RaSW vs LI−RaSW** | | | | | |
| Direct estimate | 2 | 0.34 | 0.00 | 0.98 | [ −0.66; 2.61] |
| Indirect estimate | | | | 1.06 | [ −0.12; 2.24] |
| Network estimate | | | | 1.03 | [ 0.08; 1.99] |
| **HI−RaSW vs MI−RaSW** | | | | | |
| Direct estimate | 2 | 0.34 | 0.92 | −0.12 | [ −1.73; 1.49] |
| Indirect estimate | | | | −0.79 | [ −1.94; 0.36] |
| Network estimate | | | | −0.57 | [ −1.50; 0.37] |
| **HI−RaSW vs UC** | | | | | |
| Direct estimate | 13 | 0.82 | 0.96 | 1.65 | [ 1.01; 2.29] |
| Indirect estimate | | | | 1.04 | [ −0.33; 2.41] |
| Network estimate | | | | 1.54 | [ 0.96; 2.12] |
| **HI−RaSW + CPT vs LI−RaSW** | | | | | |
| Direct estimate | 1 | 0.29 | | 0.69 | [ −1.60; 2.98] |
| Indirect estimate | | | | 1.41 | [ −0.04; 2.86] |
| Network estimate | | | | 1.20 | [ −0.02; 2.43] |
| **HI−RaSW + CPT vs UC** | | | | | |
| Direct estimate | 4 | 0.77 | 0.96 | 1.61 | [ 0.43; 2.80] |
| Indirect estimate | | | | 2.03 | [ −0.12; 4.19] |
| Network estimate | | | | 1.71 | [ 0.67; 2.75] |
| **HI−RaSW + TCM vs UC** | | | | | |
| Direct estimate | 3 | 0.69 | 0.97 | 1.88 | [ 0.57; 3.19] |
| Indirect estimate | | | | 3.89 | [ 1.92; 5.87] |
| Network estimate | | | | 2.50 | [ 1.41; 3.59] |
| **LI−FoSW vs MI−FoSW** | | | | | |
| Direct estimate | 1 | 0.59 | | −1.39 | [ −3.70; 0.91] |
| Indirect estimate | | | | −1.38 | [ −4.13; 1.36] |
| Network estimate | | | | −1.39 | [ −3.16; 0.38] |
| **LI−FoSW vs UC** | | | | | |
| Direct estimate | 1 | 0.54 | | 0.06 | [ −2.37; 2.48] |
| Indirect estimate | | | | 0.04 | [ −2.60; 2.69] |
| Network estimate | | | | 0.05 | [ −1.73; 1.84] |
| **LI−RaSW vs MI−RaSW** | | | | | |
| Direct estimate | 2 | 0.41 | 0.72 | −1.11 | [ −2.74; 0.52] |
| Indirect estimate | | | | −1.94 | [ −3.28; −0.59] |
| Network estimate | | | | −1.60 | [ −2.64; −0.56] |
| **LI−RaSW vs MI−RaSW (Acupoint)** | | | | | |
| Direct estimate | 1 | 0.33 | | −1.54 | [ −3.84; 0.75] |
| Indirect estimate | | | | −2.16 | [ −3.79; −0.54] |
| Network estimate | | | | −1.96 | [ −3.28; −0.63] |
| **LI−RaSW vs MI−RaSW + CPT** | | | | | |
| Direct estimate | 2 | 0.39 | 0.91 | −1.94 | [ −3.57; −0.31] |
| Indirect estimate | | | | −0.61 | [ −1.91; 0.69] |
| Network estimate | | | | −1.13 | [ −2.14; −0.11] |
| **LI−RaSW vs UC** | | | | | |
| Direct estimate | 2 | 0.28 | 0.98 | 0.49 | [ −1.15; 2.12] |
| Indirect estimate | | | | 0.51 | [ −0.50; 1.52] |
| Network estimate | | | | 0.51 | [ −0.35; 1.37] |
| **LI−RaSW + CPT vs MI−RaSW + CPT** | | | | | |
| Direct estimate | 1 | 0.48 | | −1.46 | [ −3.83; 0.91] |
| Indirect estimate | | | | −0.18 | [ −2.43; 2.08] |
| Network estimate | | | | −0.79 | [ −2.42; 0.84] |
| **LI−RaSW + CPT vs UC** | | | | | |
| Direct estimate | 2 | 0.87 | 0.77 | 1.22 | [ −0.45; 2.89] |
| Indirect estimate | | | | −1.58 | [ −5.82; 2.65] |
| Network estimate | | | | 0.84 | [ −0.71; 2.39] |
| **MI−FoSW vs MI−FoSW + TCM** | | | | | |
| Direct estimate | 1 | 0.47 | | −1.04 | [ −3.35; 1.27] |
| Indirect estimate | | | | 1.19 | [ −0.99; 3.37] |
| Network estimate | | | | 0.14 | [ −1.44; 1.73] |
| **MI−FoSW vs UC** | | | | | |
| Direct estimate | 3 | 0.82 | 0.95 | 1.56 | [ 0.23; 2.88] |
| Indirect estimate | | | | 0.93 | [ −1.88; 3.73] |
| Network estimate | | | | 1.44 | [ 0.24; 2.64] |
| **MI−FoSW + TCM vs UC** | | | | | |
| Direct estimate | 3 | 0.93 | 0.42 | 1.03 | [ −0.30; 2.35] |
| Indirect estimate | | | | 5.12 | [ 0.15; 10.10] |
| Network estimate | | | | 1.30 | [ 0.02; 2.58] |
| **MI−RaSW vs MI−RaSW (Acupoint)** | | | | | |
| Direct estimate | 2 | 0.57 | 0.96 | −1.29 | [ −2.90; 0.32] |
| Indirect estimate | | | | 0.87 | [ −0.98; 2.72] |
| Network estimate | | | | −0.35 | [ −1.57; 0.86] |
| **MI−RaSW vs UC** | | | | | |
| Direct estimate | 5 | 0.60 | 0.97 | 2.79 | [ 1.72; 3.86] |
| Indirect estimate | | | | 1.09 | [ −0.22; 2.39] |
| Network estimate | | | | 2.11 | [ 1.28; 2.93] |
| **MI−RaSW (Acupoint) vs UC** | | | | | |
| Direct estimate | 1 | 0.30 | | 1.01 | [ −1.27; 3.28] |
| Indirect estimate | | | | 3.09 | [ 1.59; 4.58] |
| Network estimate | | | | 2.46 | [ 1.21; 3.71] |
| **MI−RaSW + CPT vs MI−RaSW + TCM** | | | | | |
| Direct estimate | 1 | 0.50 | | −0.95 | [ −3.23; 1.33] |
| Indirect estimate | | | | 3.92 | [ 1.66; 6.18] |
| Network estimate | | | | 1.50 | [ −0.10; 3.11] |
| **MI−RaSW + CPT vs UC** | | | | | |
| Direct estimate | 7 | 0.80 | 0.89 | 1.61 | [ 0.73; 2.49] |
| Indirect estimate | | | | 1.72 | [ −0.02; 3.46] |
| Network estimate | | | | 1.63 | [ 0.85; 2.42] |
| **MI−RaSW + TCM vs UC** | | | | | |
| Direct estimate | 2 | 0.87 | 0.99 | −0.43 | [ −2.06; 1.20] |
| Indirect estimate | | | | 4.04 | [ −0.26; 8.34] |
| Network estimate | | | | 0.13 | [ −1.39; 1.65] |

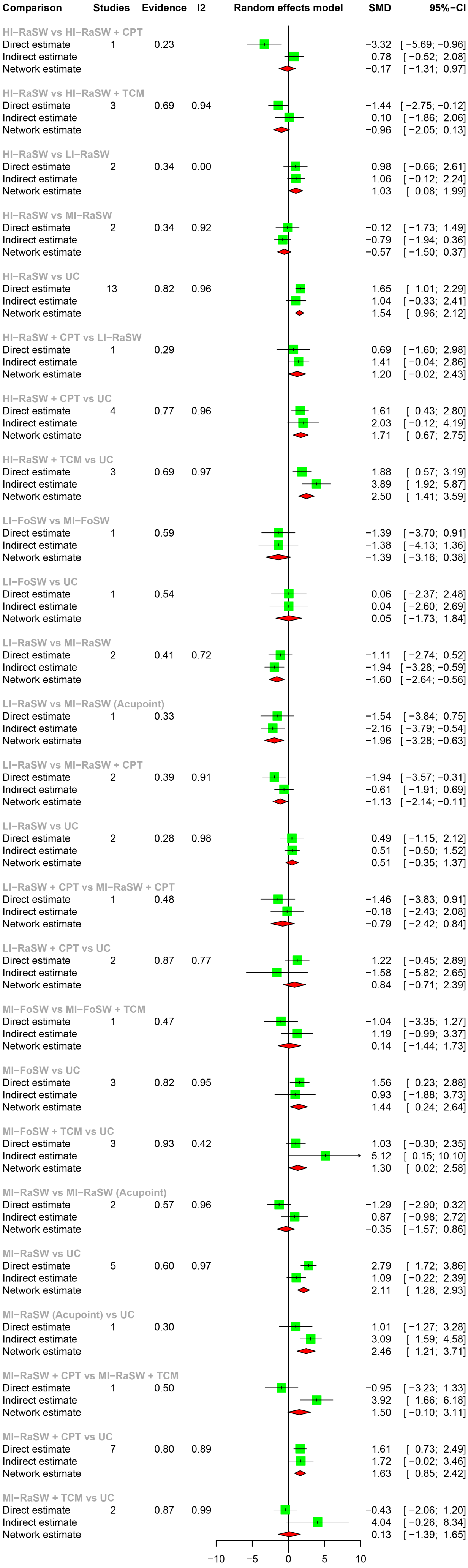

# Figure S5

| Comparison | Number of Studies | Direct Evidence | I2 | SMD | 95%-CI |
|---|---|---|---|---|---|
| **HI−FoSW vs HI−RaSW** | | | | | |
| Direct estimate | 0 | 0 | | | |
| Indirect estimate | | | | −4.13 | [ −6.46; −1.79] |
| Network estimate | | | | −4.13 | [ −6.46; −1.79] |
| **HI−FoSW vs HI−RaSW + TCM** | | | | | |
| Direct estimate | 0 | 0 | | | |
| Indirect estimate | | | | −4.09 | [ −7.08; −1.10] |
| Network estimate | | | | −4.09 | [ −7.08; −1.10] |
| **HI−FoSW vs MI−RaSW** | | | | | |
| Direct estimate | 0 | 0 | | | |
| Indirect estimate | | | | −4.07 | [ −7.26; −0.88] |
| Network estimate | | | | −4.07 | [ −7.26; −0.88] |
| **HI−FoSW vs MI−RaSW (Acupoint)** | | | | | |
| Direct estimate | 0 | 0 | | | |
| Indirect estimate | | | | −4.27 | [ −7.45; −1.08] |
| Network estimate | | | | −4.27 | [ −7.45; −1.08] |
| **HI−FoSW vs MI−RaSW (Acupoint) + TCM** | | | | | |
| Direct estimate | 0 | 0 | | | |
| Indirect estimate | | | | −2.80 | [ −6.86; 1.26] |
| Network estimate | | | | −2.80 | [ −6.86; 1.26] |
| **HI−FoSW vs MI−RaSW + CPT** | | | | | |
| Direct estimate | 0 | 0 | | | |
| Indirect estimate | | | | −4.22 | [ −7.41; −1.04] |
| Network estimate | | | | −4.22 | [ −7.41; −1.04] |
| **HI−FoSW vs MI−RaSW + TCM** | | | | | |
| Direct estimate | 0 | 0 | | | |
| Indirect estimate | | | | −3.62 | [ −7.67; 0.44] |
| Network estimate | | | | −3.62 | [ −7.67; 0.44] |
| **HI−FoSW vs UC** | | | | | |
| Direct estimate | 2 | 1.00 | 0.99 | −5.01 | [ −6.98; −3.03] |
| Indirect estimate | | | | | |
| Network estimate | | | | −5.01 | [ −6.98; −3.03] |
| **HI−RaSW vs HI−RaSW + TCM** | | | | | |
| Direct estimate | 1 | 0.81 | | 0.47 | [ −2.02; 2.95] |
| Indirect estimate | | | | −1.86 | [ −7.05; 3.33] |
| Network estimate | | | | 0.03 | [ −2.21; 2.28] |
| **HI−RaSW vs MI−RaSW** | | | | | |
| Direct estimate | 0 | 0 | | | |
| Indirect estimate | | | | 0.06 | [ −2.74; 2.85] |
| Network estimate | | | | 0.06 | [ −2.74; 2.85] |
| **HI−RaSW vs MI−RaSW (Acupoint)** | | | | | |
| Direct estimate | 0 | 0 | | | |
| Indirect estimate | | | | −0.14 | [ −2.94; 2.66] |
| Network estimate | | | | −0.14 | [ −2.94; 2.66] |
| **HI−RaSW vs MI−RaSW (Acupoint) + TCM** | | | | | |
| Direct estimate | 0 | 0 | | | |
| Indirect estimate | | | | 1.33 | [ −2.43; 5.09] |
| Network estimate | | | | 1.33 | [ −2.43; 5.09] |
| **HI−RaSW vs MI−RaSW + CPT** | | | | | |
| Direct estimate | 0 | 0 | | | |
| Indirect estimate | | | | −0.10 | [ −2.89; 2.70] |
| Network estimate | | | | −0.10 | [ −2.89; 2.70] |
| **HI−RaSW vs MI−RaSW + TCM** | | | | | |
| Direct estimate | 0 | 0 | | | |
| Indirect estimate | | | | 0.51 | [ −3.25; 4.27] |
| Network estimate | | | | 0.51 | [ −3.25; 4.27] |
| **HI−RaSW vs UC** | | | | | |
| Direct estimate | 4 | 1.00 | 0.95 | −0.88 | [ −2.13; 0.37] |
| Indirect estimate | | | | | |
| Network estimate | | | | −0.88 | [ −2.13; 0.37] |
| **HI−RaSW + TCM vs MI−RaSW** | | | | | |
| Direct estimate | 0 | 0 | | | |
| Indirect estimate | | | | 0.02 | [ −3.34; 3.38] |
| Network estimate | | | | 0.02 | [ −3.34; 3.38] |
| **HI−RaSW + TCM vs MI−RaSW (Acupoint)** | | | | | |
| Direct estimate | 0 | 0 | | | |
| Indirect estimate | | | | −0.17 | [ −3.53; 3.18] |
| Network estimate | | | | −0.17 | [ −3.53; 3.18] |
| **HI−RaSW + TCM vs MI−RaSW (Acupoint) + TCM** | | | | | |
| Direct estimate | 0 | 0 | | | |
| Indirect estimate | | | | 1.29 | [ −2.90; 5.49] |
| Network estimate | | | | 1.29 | [ −2.90; 5.49] |
| **HI−RaSW + TCM vs MI−RaSW + CPT** | | | | | |
| Direct estimate | 0 | 0 | | | |
| Indirect estimate | | | | −0.13 | [ −3.49; 3.23] |
| Network estimate | | | | −0.13 | [ −3.49; 3.23] |
| **HI−RaSW + TCM vs MI−RaSW + TCM** | | | | | |
| Direct estimate | 0 | 0 | | | |
| Indirect estimate | | | | 0.47 | [ −3.72; 4.67] |
| Network estimate | | | | 0.47 | [ −3.72; 4.67] |
| **HI−RaSW + TCM vs UC** | | | | | |
| Direct estimate | 1 | 0.81 | | −0.48 | [ −2.97; 2.00] |
| Indirect estimate | | | | −2.81 | [ −8.00; 2.38] |
| Network estimate | | | | −0.92 | [ −3.16; 1.33] |
| **MI−RaSW vs MI−RaSW (Acupoint)** | | | | | |
| Direct estimate | 0 | 0 | | | |
| Indirect estimate | | | | −0.20 | [ −3.73; 3.34] |
| Network estimate | | | | −0.20 | [ −3.73; 3.34] |
| **MI−RaSW vs MI−RaSW (Acupoint) + TCM** | | | | | |
| Direct estimate | 0 | 0 | | | |
| Indirect estimate | | | | 1.27 | [ −3.07; 5.61] |
| Network estimate | | | | 1.27 | [ −3.07; 5.61] |
| **MI−RaSW vs MI−RaSW + CPT** | | | | | |
| Direct estimate | 0 | 0 | | | |
| Indirect estimate | | | | −0.15 | [ −3.69; 3.38] |
| Network estimate | | | | −0.15 | [ −3.69; 3.38] |
| **MI−RaSW vs MI−RaSW + TCM** | | | | | |
| Direct estimate | 1 | 1.00 | | 0.45 | [ −2.06; 2.96] |
| Indirect estimate | | | | | |
| Network estimate | | | | 0.45 | [ −2.06; 2.96] |
| **MI−RaSW vs UC** | | | | | |
| Direct estimate | 1 | 1.00 | | −0.94 | [ −3.44; 1.56] |
| Indirect estimate | | | | | |
| Network estimate | | | | −0.94 | [ −3.44; 1.56] |
| **MI−RaSW (Acupoint) vs MI−RaSW (Acupoint) + TCM** | | | | | |
| Direct estimate | 1 | 1.00 | | 1.47 | [ −1.05; 3.98] |
| Indirect estimate | | | | | |
| Network estimate | | | | 1.47 | [ −1.05; 3.98] |
| **MI−RaSW (Acupoint) vs MI−RaSW + CPT** | | | | | |
| Direct estimate | 0 | 0 | | | |
| Indirect estimate | | | | 0.04 | [ −3.50; 3.58] |
| Network estimate | | | | 0.04 | [ −3.50; 3.58] |
| **MI−RaSW (Acupoint) vs MI−RaSW + TCM** | | | | | |
| Direct estimate | 0 | 0 | | | |
| Indirect estimate | | | | 0.65 | [ −3.69; 4.99] |
| Network estimate | | | | 0.65 | [ −3.69; 4.99] |
| **MI−RaSW (Acupoint) vs UC** | | | | | |
| Direct estimate | 1 | 1.00 | | −0.74 | [ −3.24; 1.76] |
| Indirect estimate | | | | | |
| Network estimate | | | | −0.74 | [ −3.24; 1.76] |
| **MI−RaSW (Acupoint) + TCM vs MI−RaSW + CPT** | | | | | |
| Direct estimate | 0 | 0 | | | |
| Indirect estimate | | | | −1.43 | [ −5.77; 2.91] |
| Network estimate | | | | −1.43 | [ −5.77; 2.91] |
| **MI−RaSW (Acupoint) + TCM vs MI−RaSW + TCM** | | | | | |
| Direct estimate | 0 | 0 | | | |
| Indirect estimate | | | | −0.82 | [ −5.83; 4.19] |
| Network estimate | | | | −0.82 | [ −5.83; 4.19] |
| **MI−RaSW (Acupoint) + TCM vs UC** | | | | | |
| Direct estimate | 0 | 0 | | | |
| Indirect estimate | | | | −2.21 | [ −5.76; 1.34] |
| Network estimate | | | | −2.21 | [ −5.76; 1.34] |
| **MI−RaSW + CPT vs MI−RaSW + TCM** | | | | | |
| Direct estimate | 0 | 0 | | | |
| Indirect estimate | | | | 0.61 | [ −3.73; 4.94] |
| Network estimate | | | | 0.61 | [ −3.73; 4.94] |
| **MI−RaSW + CPT vs UC** | | | | | |
| Direct estimate | 1 | 1.00 | | −0.78 | [ −3.28; 1.72] |
| Indirect estimate | | | | | |
| Network estimate | | | | −0.78 | [ −3.28; 1.72] |
| **MI−RaSW + TCM vs UC** | | | | | |
| Direct estimate | 0 | 0 | | | |
| Indirect estimate | | | | −1.39 | [ −4.93; 2.15] |
| Network estimate | | | | −1.39 | [ −4.93; 2.15] |

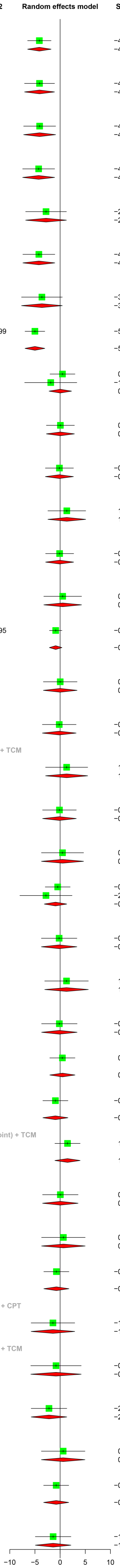

# Figure S6

## (A) Pain

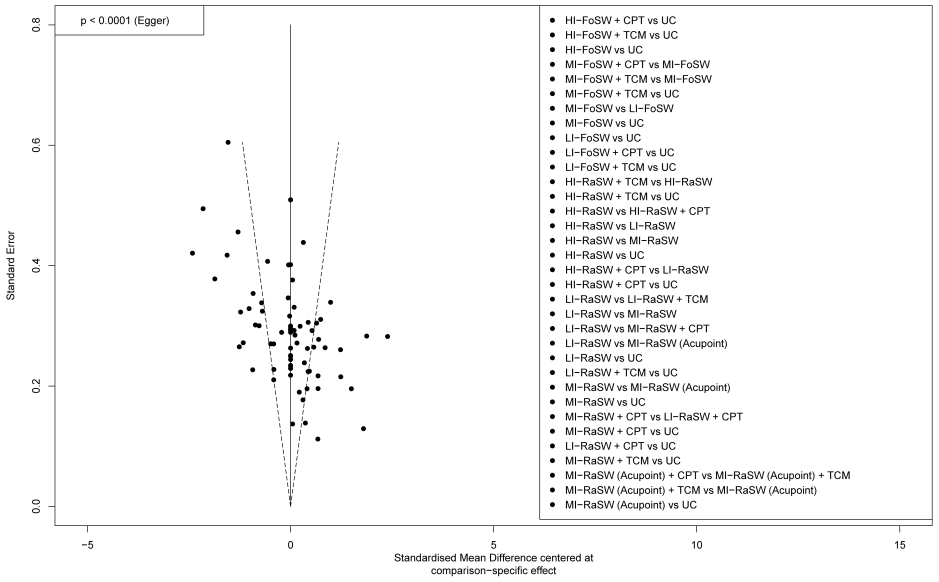


## (B) Global function

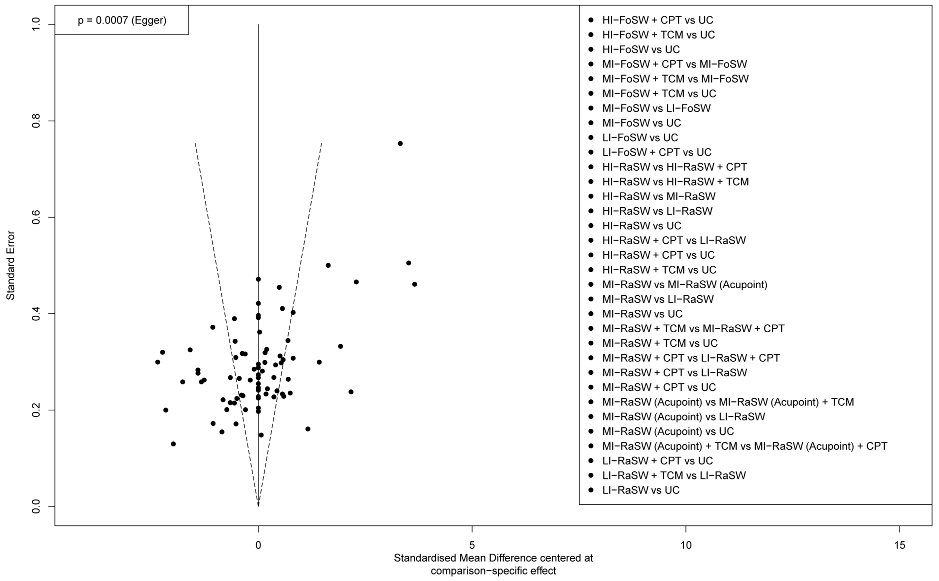


## (C) Disease inflammation

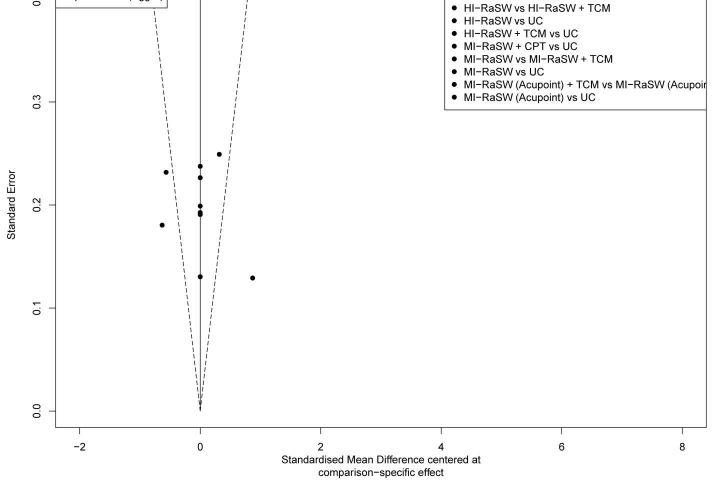

Supplement: Supplementary file 1 [file biomedicines-10-00306-s001.zip › Supplementary figures S1-S6.pdf]
