# Supplementary material for: Relative Effect of Extracorporeal Shockwave Therapy Alone or in Combination with Noninjective Treatments on Pain and Physical Function in Knee Osteoarthritis: A Network Meta-Analysis of Randomized Controlled Trials
Source: Biomedicines. 2022 Jan 28;10(2):306. doi: 10.3390/biomedicines10020306 (PMC8869515; doi:10.3390/biomedicines10020306)
Supplement: Supplementary file 1 [file biomedicines-10-00306-s001.zip › Supplementary tables S1-S9.pdf]

Supplementary materials

Table S1. Database search strategies.

Data base	Search terms for query
**Pubmed**	
#1	(((shockwave therapy) OR shock wave therapy) OR extracorporeal shockwave therapy) OR extracorporeal shock wave therapy
#2	Osteoarthritis
#3	Gonarthritis
#4	Degenerative cartilage disease
#5	((#2) OR #3) OR #4
#6	Randomized control trial
#7	#1 AND #5 AND #6
**Physiotherapy Evidence Database (PEDro)**	
	Body part: lower leg or knee
	Method: clinical trial
	Abstract & Title:
#1	extracorporeal shock wave therapy
#2	extracorporeal shockwave therapy
#3	shock wave therapy
#4	shockwave therapy
#5	shock wave
#6	shockwave
#7	Osteoarthritis
#8	Gonarthritis
#9	Degenerative cartilage disease
**Cochrane Library Database**	
#1	(shock wave) OR (shockwave)
#2	Osteoarthritis
#3	Gonarthritis
#4	Degenerative cartilage disease
#5	(knee soft tissue disorder) or (knee musculoskeletal disorder)
#6	#2 OR #3 OR #4 OR #5
#7	#1 AND #6

(continued)

Table S1. (continued)

Data base	Search terms for query
**Excerpta Medica dataBASE (EMBASE)**	
#1	extracorporeal shock wave therapy
#2	extracorporeal shockwave therapy
#3	shock wave therapy
#4	shockwave therapy
#5	#1 OR #2 OR #3 OR #4
#6	'osteoarthritis'/exp OR osteoarthritis
#7	Gonarthritis
#8	degenerative AND cartilage AND disease
#9	knee AND joint AND musculoskeletal AND disorder
#10	knee AND joint AND soft AND tissue AND disorder
#11	#6 OR #7 OR #8 OR #9 OR #10
#12	#5 AND #11
#13	(#12) AND [randomized controlled trial]/lim AND ([article]/lim OR [article in press]/lim) AND [humans]/lim
**China knowledge resource integrated database**	
#1	(shock wave therapy) OR (shock wave therapy)
#2	(knee) AND (osteoarthritis)
#3	(knee) AND (Gonarthritis)
#4	(knee) AND (Degenerative cartilage disease)
#6	(knee soft tissue disorder) or (knee musculoskeletal disorder)
#7	#2 OR #3 OR #4 OR #5 OR #6
#8	#1 AND #7
**Google Scholar**	
#1	allintitle: extracorporeal shock wave therapy osteoarthritis
#2	allintitle: extracorporeal shockwave therapy Gonarthritis
#3	allintitle: extracorporeal shock wave therapy degenerative cartilage disease

Table S2. Characteristics of the included studies.

Study (year) [Reference]	Country (area)	Study arm	Age (years) Mean (SD or range)	BMI (kg/m^2)	Sex (F/M)	*N*	Involved side, unilateral/bilateral	K-L grade	Duration of symptoms (months) Mean (SD, range)	Co-intervention	All cause withdraws (n)	Compliance rate	Measured time point (week)	Main outcome measures
Aletaibieke, 2016 [1]	China	EG: ESWT	65.0 (45–85)	31.2 (5.2)	22/21	43	15/0	I, II	NR	None	0	100.00%	0, 12	VAS; WOMAC
	(Asia)	CG: PLA	64.5 (45–89)	28.9 (3.7)	25/18	43	15/0				0	100.00%		Lequesne's index
Ammar 2018 [2]	Egypt	EG: ESWT	40.1 (5.5)	28.8 (5.2)	11/4	15	NR	NR	17.2 (11.4)	MSE + CPTM (IFC)	3	80.00%	0, 4	VAS; WOMAC
	(Africa)	CG: Con	43.6 (7.7)	29.2 (2.5)	6/9	15			18.7 (13.1)		2	86.67%		
Cai 2021 [3]	China	EG: ESWT	62.4 (2.2)	NR	16/28	44	44/0	II-IV	26.2 (4.0)	None	0	100.00%	0, 6	WOMAC
	(Asia)	CG: Con	62.3 (2.2)		15/29	44	44/0		25.9 (3.8)		0	100.00%		Inflammation factor
Chang 2021 [4]	China	EG 2: Acupoint-ESWT	59.7 (5.3)	NR	30/15	45	NR	I-III	26.6 (5.2)	None	0	100.00%	0, 2, 4	VAS; WOMAC
	(Asia)	EG 1: Acupoint-ESWT +TCM	59.0 (5.7)		29/16	45			26.3 (3.6)		0	100.00%		Lysholm scale<br>Inflammation factor
Chen 2014 [5]	Taiwan	EG: ESWT + MSE	63.0 (7.4)[c]	NR	102/28c	30	0/30	III	10–144[c]	None	2	93.33%	0, 8, 24	VAS;
	(Asia)	CG 1: USD + MSE				30	0/30				2	93.33%		Lequesne's index
		CG 2: MSE				30	0/30				3	90.00%		
		CG 3: Con				30	0/30				3	90.00%		
Cho 2016 [6]	Korea	EG: ESWT	75.5 (7.7)	22.9 (3.6)	1/8	9	2/7	≥1	NR	None	0	100.00%	0, 4	VAS; FIM
	(Asia)	CG: PLA	72.7 (5.9)	23.5 (4.3)	2/7	9	1/8	≥1			0	100.00%		
Cui 2015 [7]	China	EG 1: ESWT + TCM	56.8 (13.1)[c]	NR	45/30c	25	52/33[c]	NR	63.6 (21.6)[c]	None	0	100.00%	0, 1, 2, 4	Lequesne's index
	(Asia)	EG 2: ESWT				25					0	100.00%		
		CG: TCM				25					0	100.00%		
Dou 2016 [8]	China	EG: ESWT	51.5 (8.9)	23.1 (3.2)	50/13	63	NR	I, II	7.84 (11.31)	None	2	96.83%	0, 1, 2, 3, 4, 5	WOMAC
	(Asia)	CG: USD	52.1 (9.2)	22.7 (3.2)	44/14	58			8.23 (12.27)		0	100.00%		
Ediz 2018 [9]	Turkey	EG 1: ESWT	70.5 (4.2)	26.9 (4.7)	24/14	38	0/38	II, III	40.72 (15.14)	TENS	2	95.00%	0, 24, 52	VAS; WOMAC
	(Asia)	EG 2: PLA (LI ESWT)	69.7 (4.5)	26.9 (4.9)	22/13	35	0/35		39.43 (12.34)		5	87.50%		Lequesne's index
Eftekharsadat	Iran	EG: ESWT	58.0 (6.0)	NR	25/0	25	NR	II, III	NR	MSE	2	92.00%	0, 3, 7	VAS
2020 [10]	(Asia)	CG 1: USD	55.8 (7.2)		23/2	25					3	88.00%		WOMAC
		CG 2: Con	58.2 (7.2)		22/3	25					3	88.00%		
Elerian 2016 [11]	Egypt	EG: ESWT	51.0 (3.5)[c]	86% ≥25.0	50/10c	20	0/20	II, III	NR	None	0	100.00%	0, 4, 8, 24	VAS
	(Africa)	CG 1: IAI				20	0/20				0	100.00%		WOMAC
		CG 2: PLA				20	0/20				0	100.00%		
Elgendy 2020 [12]	Egypt	EG: ESWT	48.7 (8.6)	31.3 (2.3)	NR	15	15/0	II, III	NR	MSE + CPTM	0	100.00%	0, 4	VAS
	(Africa)	CG 1: IAI	49.2 (9.2)	31.5 (2.0)		15	15/0			(USD)	0	100.00%		WOMAC
		CG 2: Con	55.1 (6.7)	30.8 (2.5)		15	15/0				0	100.00%		

To be continued.

Table S2. Continued.

Study (year) [Reference]	Country (area)	Study arm	Age (years) Mean (SD or range)	BMI (kg/m^2)	Sex (F/M)	*N*	Involved side, unilateral/bilateral	K-L grade	Duration of symptoms (months) Mean (SD, range)	Co-intervention	All cause withdraws (n)	Compliance rate	Measured time point (week)	Main outcome measures
El-Sakka 2019 [13]	Egypt	EG: ESWT	53.5 (7.8)	35.3 (5.3)	9/6	15	15/0	NR	35.3 (30.9)	MSE	0	100.00%	0, 4, 8	VAS; WOMAC
	(Africa)	CG: USD	51.5 (5.7)	36.4 (3.1)	12/3	15	15/0		57.9 (42.1)		0	100.00%		
Feng 2019 [14]	China	EG 1: ESWT + TCM	54.5 (3.6)	NR	44/0	44	NR	NR	28.8 (6.0)	None	0	100.00%	0, 4	VAS; WOMAC
	(Asia)	EG 2: ESWT	57.2 (3.4)		44/0	44			30.0 (7.2)		0	100.00%		
Gunaydin 2020 [15]	Turkey	EG: ESWT	58.8 (6.2)[c]	28.8 (4.7)[c]	8/0	8	48/48[c]	I-III	≥6.0	MSE	10	44.44%	0, 6, 12	VAS; KOOS
	(Asia)	CG 1: KT			20/0	20					2	90.91%		
		CG 2: Con			20/0	20					0	100.00%		
Guo 2019 [16]	China	EG: ESWT	59.0 (8.3)	NR	27/16	43	43/0	II, III	50.5 (39.0)	TCM	0	100.00%	0, 1.5	VAS
	(Asia)	CG: Con	59.0 (8.5)		25/15	40	40/0		28.8 (6.0)		3	93.02%		
Hammam 2020 [17]	Egypt	EG 1: HI ESWT	50.4 (3.4)	30.7 (3.5)	9/6	15	15/0	II	≥3.0	MSE	0	100.00%	0, 4	VAS
	(Africa)	EG 2: LI ESWT	49.9 (2.6)	31.0 (2.4)	10/5	15	15/0				0	100.00%		KOOS
		CG: PLA	49.7 (3.1)	31.1 (3.0)	7/8	15	15/0				0	100.00%		
He 2014 [18]	China	EG: ESWT	50.7 (9.1)	NR	16/8	24	17/7	NR	6.1 (6.4)	CPTM	0	100.00%	0, 4	KSS
	(Asia)	CG: Con	52.3 (10.3)		13/11	24	20/4		6.1 (6.4)		0	100.00%		
He 2016 [19]	China	EG: ESWT	65.0[c]	NR	106/35c	68	NR	NR	≥1.0	None	0	100.00%	0, 6, 14, 26	VAS
	(Asia)	CG: Con				73					0	100.00%		Lysholm scale
He 2021 [20]	China	EG: ESWT	53.0 (5.0)	22.3 (2.0)	30/31	61	NR	II, III	51.0 (5.0)	PM (NSAID)	0	100.00%	0, 6	WOMAC; inflammation factor
	(Asia)	CG: Con	53.0 (5.0)	22.3 (2.0)	30/31	61			51.0 (5.0)		0	100.00%		Lequesne's index
Imamura 2017 [21]	Brazil	EG: ESWT	70.0 (6.5)	NR	52/0	52	52/0	II-IV	92.4 (77.4)	None	3	94.23%	0, 4, 12	VAS; WOMAC
	(Europe)	CG: PLA	72.4 (6.5)		53/0	53	53/0		113.7 (99.0)		2	96.23%		
Ji 2021 [22]	China	EG: Acupoint-ESWT	69.9 (5.3)	NR	32/22	54	54/0	NR	13.5 (4.4)	None	0	100.00%	0, 12, 36	VAS; Lysholm scale
	(Asia)	CG: PM (NSAID)	70.4 (6.7)		34/20	54	54/0		15.0 (3.9)		0	100.00%		inflammation factor
Jiang 2017 [23]	China	EG 1: ESWT + Mobilization	56.2 (4.9)	NR	13/12	25	NR	II, III	≥6.0	Regular medicine	0	100.00%	0, 4	WOMAC
	(Asia)	EG 2: ESWT	59.1 (3.1)		14/11	25					0	100.00%		
		CG: Mobilization	57.3 (4.7)		12/13	25					0	100.00%		
Kim 2015 [24]	Korea	EG 1: HI ESWT	63.5 (5.4)	24.2 (2.1)	3/27	30	30/0	II, III	16.7 (4.7)	None	0	100.00%	0, 1, 4, 12	VAS
	(Asia)	EG 2: LI ESWT	65.1 (6.3)	23.8 (1.7)	4/26	30	30/0		19.3 (5.2)		0	100.00%		WOMAC
														Lequesne's index

To be continued.

Table S2. Continued.

Study (year) [Reference]	Country (area)	Study arm	Age (years) Mean (SD or range)	BMI (kg/m^2)	Sex (F/M)	*N*	Involved side, unilateral/bilateral	K-L grade	Duration of symptoms (months) Mean (SD, range)	Co-intervention	All cause withdraws (n)	Compliance rate	Measured time point (week)	Main outcome measures
Kuang 2017 [25]	China	EG 1: ESWT + TCM	65.8 (61-75)	NR	19/11	30	NR	I, II	88.8 (9.6-228)	None	0	100.00%	0, 5	VAS; WOMAC
	(Asia)	EG 2: ESWT	66.4 (60-74)		18/12	30			91.2 (6.0-240)		0	100.00%		
Li 2018 [26]	China	EG: ESWT + TCM	51.8 (11.7)	NR	99/68	167	NR	I-III	57.0 (50.9)	None	0	100.00%	0, 5, 12, 24	VAS; WOMAC
	(Asia)	CG: PM (NSAID)	52.4 (12.3)		108/59	167			49.8 (46.3)		0	100.00%		
Li 2015 [27]	China	EG 1: HF-ESWT	53 (46–69)	NR	19/11	30	NR	I-III	61.2 (36–84)	None	0	100.00%	0, 2	VAS
	(Asia)	EG 2: MF-ESWT	55 (48–70)		17/13	30			70.8 (36–108)		0	100.00%		
		EG 3: LF-ESWT	52 (45–70)		18/12	30			62.4 (36–96)		0	100.00%		
		CG: Conventional acupucture	54 (46–69)		18/12	30			61.2 (36–84)		0	100.00%		
Li MZ 2020 [28]	China	EG: ESWT	57.4 (9.1)	23.7 (2.8)	17/1	18	NR	II, III	≥6.0	MSE	0	100.00%	0, 4, 16	VAS; WOMAC
	(Asia)	CG: PLA	58.7 (13.0)	24.5 (3.2)	16/2	18					0	100.00%		
Li RX 2021 [29]	China	EG: ESWT	NR	NR	NR	35	NR	NR	NR	None	0	100.00%	0, 4	VAS; WOMAC
	(Asia)	CG: MSE				35					0	100.00%		Lequesne's index
Li 2017 [30]	China	EG: ESWT	64.3 (11.8)	27.3 (2.5)	45/0	45	42/3	II, III	2.0-24.0[c]	None	3	93.33%	0, 8, 16	VAS; WOMAC
	(Asia)	CG: PLA + PM (NSAID)	65.4 (12.7)	26.2 (2.8)	45/0	45	42/3				2	95.56%		Lequesne's index
Liu 2020 [31]	China	EG 1: HI ESWT	58.6 (6.6)	22.9 (1.3)	14/12	26	22/4	I-III	61.4 (32.9)	None	0	100.00%	0, 8, 56	VAS; WOMAC
	(Asia)	EG 2: MI ESWT	58.5 (5.3)	22.5 (1.4)	37/38	75	60/15		62.7 (32.4)		0	100.00%		
		EG 3: LI ESWT	58.2 (5.7)	22.6 (1.3)	14/12	26	22/4		62.0 (31.8)		0	100.00%		Lysholm scale
Liu MY 2017 [32]	China	EG: ESWT	68.5 (7.6)	26.4 (2.5)	65/14	79	79/0	II, III	25.8 (3.5)	None	0	100.00%	0, 8, 16	VAS; WOMAC
	(Asia)	CG: PLA + PM (NSAID)	66.7 (7.4)	25.8 (3.1)	61/18	79	79/0		26.8 (3.6)		0	100.00%		Inflammation factor
Liu WD 2017 [33]	China	EG: ESWT	59.1 (5.3)	NR	17/13	30	NR	I-III	25.2 (7.1)	None	0	100.00%	0, 6	WOMAC
	(Asia)	CG: Acupotomy	58.8 (5.1)		17/11	28			24.7 (6.2)		2	93.33%		
Liu 2016b [34]	China	EG: ESWT	59.6 (8.2)	26.8 (3.0)	33/0	33	33/0	II, III	58.8 (39.6)	None	0	100.00%	0, 8	VAS; WOMAC
	(Asia)	CG: GAS	58.7 (6.4)	27.4 (2.6)	32/0	32	32/0		61.2 (40.8)		0	100.00%		
Liu YW 2018 [35]	China	EG: ESWT + TCM	51.8 (7.2)	NR	67/13c	40	38/42[c]	NR	21.4 (9.4)	None	0	100.00%	0, 3, 7	WOMAC
	(Asia)	CG: PM (NSAID)	52.9 (6.6)			40			20.9 (8.9)		0	100.00%		HSS
Lizis 2017a [36]	Poland	EG: ESWT	63.5 (8.0)	24.9 (1.9)	13/7	20	20/0	NR	108 (36)	Glucocorticoids	0	100.00%	0, 5	WOMAC
	(Europe)	CG: MSE	65.0 (8.4)	24.1 (1.5)	9/11	20	20/0		138 (72)		0	100.00%		
Lizis 2017b [37]	Poland	EG: ESWT	61.0 (9.0)	23.2 (1.3)	30/0	30	0/30	NR	96 (48)	None	0	100.00%	0, 5	VAS; WOMAC
	(Europe)	CG: MSE	59.0 (9.0)	23.3 (1.3)	30/0	30	0/30		84 (48)		0	100.00%		

To be continued.

Table S2. Continued.

Study (year) [Reference]	Country (area)	Study arm	Age (years) Mean (SD or range)	BMI (kg/m^2)	Sex (F/M)	*N*	Involved side, unilateral/bilateral	K-L grade	Duration of symptoms (months) Mean (SD, range)	Co-intervention	All cause withdraws (n)	Compliance rate	Measured time point (week)	Main outcome measures
Lizis 2018 [38]	Poland	EG: ESWT	59.8 (3.9)	24.2 (0.7)	10/20	30	30/0	≥1	96 (24)	None	0	100.00%	0, 5	VAS; KOOS
	(Europe)	CG: USD	60.7 (4.8)	23.8 (0.7)	8/22	30	30/0		88.8 (28.8)		0	100.00%		
Ma 2020 [39]	China	EG: ESWT	58.4 (2.6)	NR	NR	38	38/0	II, III	55.9 (13.8)	TCM (fumigation)	0	100.00%	0, 2	VAS
	(Asia)	CG: Con	58.4 (2.6)			38	38/0		54.1 (14.4)		0	100.00%		
Qin 2018 [40]	China	EG: ESWT	58.3 (10.3)	NR	17/13	30	NR	NR	38.4 (7.2)	None	0	100.00%	0, 4	VAS
	(Asia)	CG: Phonophoresis	56.2 (11.7)		19/11	30			46.8 (6.0)		0	100.00%		
Shen 2016 [41]	China	EG: ESWT	61.1 (8.8)	22.8 (3.5)[d]	17/14	31	31/0	II, III	56.3 (21.4)	None	0	100.00%	0, 4	VAS;
	(Asia)	CG: CPTM + TCM	62.5 (8.3)	23.3 (3.6)[d]	19/11	30	30/0		47.0 (19.9)		0	100.00%		Lysholm scale
Shenouda 2013 [42]	Egypt	EG: ESWT	52.2 (5.4)	NR	NR	15	45/45[c]	III, IV	88.0 (33.2)	MSE	0	100.00%	0, 4	VAS
	(Africa)	CG 1: MWM	51.9 (6.5)			15			100.8 (29.6)		0	100.00%		WOMAC
		CG 2: Con	50.1 (5.7)			15			92.8 (33.7)		0	100.00%		
Shi D 2020 [43]	China	EG: ESWT + TCM	59.6 (8.1)	NR	18/12	30	30/0	NR	39.4 (10.4)	None	0	100.00%	0, 4, 8, 16, 28	VAS; WOMAC
	(Asia)	CG: GAS	56.8 (10.1)		21/9	30	30/0		40.1 (11.3)		0	100.00%		
Uysal 2020 [44]	Turkey	EG 1: ESWT	60.2 (6.3)	30.6 (4.3)	42/10	52	NR	II, III	40.2 (21.9)	MSE + CPTM	0	100.00%	0, 4, 12	VAS; WOMAC
	(Asia)	EG 2: PLA (LI ESWT)	61.8 (6.0)	30.8 (4.6)	43/9	52			46.8 (24.0)	(TENS)	0	100.00%		Lequesne's index
Wang AH 2021 [45]	China	EG: ESWT	65.2 (5.4)	NR	29/13	42	NR	I, II	19.4 (17.1)	MSE	0	100.00%	0, 4	JOA
	(Asia)	CG: Con	58.4 (2.6)		30/11	41			18.2 (16.9)		0	100.00%		
Wang HR 2020 [46]	China	EG 1: ESWT + TCM	59.6 (9.5)	NR	56/64	120	95/25	I-III	59.6 (20.2)	None	0	100.00%	0, 2, 6, 12, 24	VAS
	(Asia)	EG 2: ESWT	58.3 (15.3)		61/59	120	88/32		56.0 (20.2)		0	100.00%		Lysholm scale
		CG: TCM	60.8 (10.5)		55/65	120	92/28		54.7 (19.2)		0	100.00%		Inflammation factor
Wang 2016 [47]	China	EG: ESWT	63.5 (9.5)	NR	19/21	40	19/21	NR	106.8 (91.2)	None	0	100.00%	0, 8, 19, 32	WOMAC
	(Asia)	CG: Manipulation	64.2 (9.6)		18/20	38	17/21		103.2 (92.4)		0	100.00%		
Wang TS 2020 [48]	China	EG: ESWT	63.5 (10.6)	24.8 (2.3)	12/24	36	NR	NR	97.2 (42.0)	None	3	91.67%	0, 5, 10	VAS; WOMAC
	(Asia)	CG: PLA	64.3 (11.1)	25.2 (2.4)	15/21	36			92.4 (45.6)		5	86.11%		
Wang YY 2020 [49]	China	EG 1: Acupoint-ESWT	80.3 (10.0)	NR	17/22	39	NR	NR	136.8 (56.4)	None	0	100.00%	0, 4, 8	WOMAC
	(Asia)	EG 2: ESWT	78.5 (10.9)		23/13	36			136.8 (52.8)		0	100.00%		
Wu 2014 [50]	China	EG: ESWT	59.7 (6.9)	23.9 (1.8)	20/7	27	27/0	II, III	NR	None	2	92.59%	0, 2, 6	VAS
	(Asia)	CG: GAS + PM (NSAID)	60.6 (8.5)	23.8 (1.8)	20/6	26	26/0				5	80.77%		KOOS
Xiao 2021 [51]	China	EG: ESWT	62.9 (8.9)	NR	24/34	58	NR	I-III	46.7 (10.8)	MSE	0	100.00%	0, 4	Inflammation
	(Asia)	CG: Con	63.2 (8.8)		22/36	58			48.6 (11.2)	PM (NSAID)	0	100.00%		factor

To be continued.

Table S2. Continued.

Study (year) [Reference]	Country (area)	Study arm	Age (years) Mean (SD or range)	BMI (kg/m^2)	Sex (F/M)	*N*	Involved side, unilateral/bilateral	K-L grade	Duration of symptoms (months) Mean (SD, range)	Co-intervention	All cause withdraws (n)	Compliance rate	Measured time point (week)	Main outcome measures
Xie 2019 [52]	China	EG 1: ESWT + TCM	57.0 (11.6)	NR	30/0	30	29/1	II, III	15.4 (5.4)	None	0	100.00%	0, 4, 16	VAS
	(Asia)	EG 2: ESWT	55.8 (10.9)		30/0	30	26/4		16.9 (6.0)		0	100.00%		Lequesne's index
		CG: TCM (fumigation)	56.4 (11.2)		30/0	30	28/2		15.7 (6.1)		0	100.00%		
Xing 2018 [53]	China	EG: ESWT	60.2 (10.1)	NR	11/14	25	25/0	I, II	7.5 (3.0-120)	PM (Diclofenac	0	100.00%	0, 4	VAS
	(Asia)	CG: Con	59.7 (9.8)		9/15	24	24/0		7.8 (2.0-96)	Diethylamine Emulgel)	0	100.00%		Lysholm scale
Yang 2017 [54]	China	EG 1: ESWT + TCM (fumigation)	63.9 (9.9)	NR	41/3	44	NR	NR	NR	MSE	0	100.00%	0, 4	WOMAC
	(Asia)	EG 2: ESWT	61.6 (11.1)		38/3	41				PM	0	100.00%		
		CG: TCM (fumigation)	66.3 (9.7)		40/5	45					0	100.00%		
Yang 2021 [55]	China	EG: ESWT	57.8 (6.5)	NR	23/14	37	NR	I-III	29.8 (7.1)	MSE	0	100.00%	0, 4	VAS; WOMAC
	(Asia)	CG: Con	58.3 (7.2)		25/12	37			28.6 (6.7)	Acupuncture	0	100.00%		
Yu 2008 [56]	China	EG: ESWT	64.9 (8.1)	27.2 (5.0)	26/11	37	37/0	I, II		None	5	88.10%	0, 7, 11, 15	VAS; KSS
	(Asia)	CG: PLA	64.8 (7.3)	27.2 (5.0)	24/11	35	35/0				7	83.33%		Lequesne's index
Yu 2019 [57]	China	EG 1: Acupoint-ESWT	65.6 (4.3)	NR	46/29	75	NR	I-III	66.4 (12.8)	None	0	100.00%	0, 4, 8, 12	WOMAC
	(Asia)	EG 2: ESWT	64.4 (4.3)		44/31	75			66.1 (12.1)		0	100.00%		
Zhang 2017 [58]	China	EG: ESWT	52.6 (9.2)	NR	30/25	55	NR	NR	NR	MSE	0	100.00%	0, 4, 5, 8	VAS
	(Asia)	CG: CPTM (USD)	53.2 (9.5)		28/23	51					0	100.00%		Lysholm scale
Zhang XG 2016 [59]	China	EG 1: Acupoint-ESWT	63.6 (5.5)	NR	25/13	38	26/12	I-III	194.5 (92.5)	None	0	100.00%	0, 2, 6, 14	VAS
	(Asia)	EG 2: ESWT	63.9 (5.6)		22/15	37	24/13		200.2 (77.7)		0	100.00%		Lysholm scale
Zhang Y 2016 [60]	China	EG: ESWT + TCM (fumigation)	61.8 (5.3)[c]	NR	46/34c	40	62/18[c]	II, III	8.0-372[c]	None	0	100.00%	0, 3	Lequesne's index
	(Asia)	CG: GAS + PM				40					0	100.00%		
Zhang 2020 [61]	China	EG: ESWT	53.2 (5.0)	22.6 (2.0)	22/11	33		I-III	51.3 (22.4)	PM (NSAID)	2	94.29%	0, 6	WOMAC
	(Asia)	CG: Con	54.3 (5.3)	23.0 (2.5)	23/11	34			55.4 (21.5)		1	97.14%		Lequesne's index; Inflammation factor
Zhang 2021 [62]	China	EG 1: HI ESWT	60.9 (8.7)[d]	24.9 (2.1)[d]	22/15	37	37/0	II, III	17.6 (6.3)[d]	None	0	100.00%	0, 2, 4, 8	VAS; WOMAC
	(Asia)	EG 2: MI ESWT	61.8 (7.9)[d]	24.6 (1.6)[d]	23/15	38	38/0		18.5 (6.2)[d]		0	100.00%		
		EG 3: PLA (LI ESWT)	61.5 (5.4)	25.0 (1.3)	8/6	14	14/0		15.7 (8.4)		0	100.00%		
Zhao 2016 [63]	China	EG: ESWT	52.4 (45-68)[c]	NR	22/38c	30	NR	II, III	28.8 (4.0-72.0)[c]	GAS, PM (NSAID)	0	100.00%	0, 8	VAS; WOMAC
	(Asia)	CG: PLA				30					0	100.00%		Lequesne's index

To be continued.

Table S2. Continued.

Study (year) [Reference]	Country (area)	Study arm	Age (years) Mean (SD or range)	BMI (kg/m^2)	Sex (F/M)	*N*	Involved side, unilateral/bilateral	K-L grade	Duration of symptoms (months) Mean (SD, range)	Co-intervention	All cause withdraws (n)	Compliance rate	Measured time point (week)	Main outcome measures
Zhao 2013 [64];	China	EG: ESWT	59.9 (11.3)	26.1 (2.6)	20/14	34	NR	II, III	≥3.0	None	4	88.24%	0, 1, 4, 12, 16	VAS; WOMAC
2014 [65]	(Asia)	CG: PLA	61.8 (9.8)	25.6 (2.5)	25/11	36					5	86.11%		Lequesne's index
Zheng 2021 [66]	China	EG: ESWT	62.3 (1.2)	25.3 (0.2)	24/23	47	NR	II, III	23.1 (2.4)	None	0	100.00%	0, 5	VAS;
	(Asia)	CG: PM (NSAID)	62.2 (1.4)	25.2 (0.4)	25/22	47			23.2 (2.6)		0	100.00%		Lequesne's index
														Inflammation factor
Zhong 2019 [67]	China	EG 1: ESWT	62.5 (8.2)	25.3 (2.3)	21/11	32	NR	II, III	34.7 (15.4)	MSE	2	93.75%	0, 5, 12	VAS; WOMAC
	(Asia)	EG 2: PLA (LI ESWT)	63.2 (7.7)	25.4 (2.9)	19/12	31			34.1 (14.2)		1	96.77%		Lequesne's index
Zhou 2019 [68]	China	EG 1: ESWT + MSE	69.9 (4.7)	NR	26/16	42	NR	NR	15.6 (6.0)	None	0	100.00%	0, 1	VAS; WOMAC
	(Asia)	EG 2: ESWT	69.7 (4.6)		27/15	42			14.4 (7.2)		0	100.00%		Lequesne's index
Zhu CY 2021 [69]	China	EG 1: Acupoint_ESWT + TCM	46.9 (11.2)	NR	31/25	56	NR	NR	49.4 (22.9)	MSE	0	100.00%	0, 8	VAS
	(Asia)	EG 2: Acupoint_ESWT	47.8 (10.1)		29/27	56			48.1 (19.9)		0	100.00%		JOA
Zhu HM 2021 [70]	China	EG 1: ESWT + Acupuncture	58.9 (13.6)	NR	24/16	40	NR	I-III	12.3 (5.6)	None	0	100.00%	0, 2	WOMAC
	(Asia)	EG 2: ESWT + PM (NSAID)	59.9 (13.8)		23/17	40			12.9 (4.1)		0	100.00%		

Con, control; CPTM, conventional physical therapy modality; ESWT, extracorporeal shockwave therapy; FIM, Functional Independence Measure; GAS, glucosamine ammonia sugar; HSS, Hospital for Special Surgery; IFC, interferential current; JOA, Japanese orthopaedics association; KOOS, Knee Injury and Osteoarthritis Outcome Score; KSS, Knee Society Score; KT, kinesio taping; MWM, mobilization with movement; NSAID, non-steroidal anti-inflammatory drug; PLA, placebo; PM, pain medication; TENS, transcutaneous electrical nerve stimulation; USD, ultrasound diathermy.

Reference

1. Aletaibieke, N.; Zhang, Y.L.; Sun, K.J.; Li, K.X. Clinical efficacy of extracorporeal shockwave therapy for treating early and middle stage of knee osteoarthritis. *J Nongken Med.* **2016**, 38, 434-436.
2. Ammar, T. Shock wave therapy versus interferential therapy in knee osteoarthritis. *Int J Physiother Res.* **2018**, 6, 2771-2776.
3. Cai, C.Y.; Liang, X.D.; Shan, H.X. Analysis on efficacy of extracorporeal shock wave combined with glucosamine in the treatment of knee osteoarthritis. *Youjiang Med J.* **2021**, 49, 126-129.
4. Chang, Y.W.; Ji, J.G. Effect of radial extracorporeal shock wave acupoint therapy combined with Duhuo Jisheng Decoction on lower limb function in elderly patients with knee osteoarthritis. *J Hubei Uni Chin Med.* **2021**, 23, 100-103.
5. Chen, T.W.; Lin, C.W.; Lee, C.L.; Chen, C.H.; Chen, Y.J.; Lin, T.Y.; Huang, M.H. The efficacy of shock wave therapy in patients with knee osteoarthritis and popliteal cyamella. *Kaohsiung J Med Sci.* **2014**, 30, 362-370.
6. Cho, S.J.; Yang, J.R.; Yang, H.S.; Yang, H.E. Effects of Extracorporeal Shockwave Therapy in Chronic Stroke Patients With Knee Osteoarthritis: A Pilot Study. *Ann Rehabil Med.* **2016**, 40, 862-870.
7. Cui, X.Q. Twenty-Five Cases of Knee Osteoarthritis Treated with Extracorporeal Shock Wave in Combination with Zishen Gukang Pill. *Henan Tradit Chin Med.* **2015**, 35, 1323-1324.
8. Dou, Y.S.; Yuan, J.; Zhao, J.Y. Investigation of short-term efficacy of extracorporeal shock wave therapy for knee osteoarthritis. *Chin J Rehabil Med.* **2016**, 31, 917-919.
9. Ediz, L.; Özgokce, M. Effectiveness of Extracorporeal Shock Wave Therapy to Treat Primary Medial Knee Osteoarthritis with and without Bone Marrow Edema in Elderly Patients. *Turk J Geriatri.* **2018**, 21, 394-401.

10. Eftekharsadat, B.; Jahanjoo, F.; Toopchizadeh, V.; Heidari, F.; Ahmadi, R.; Babaei-Ghazani, A. Extracorporeal shockwave therapy and physiotherapy in patients with moderate knee osteoarthritis. *Crescent J Med Biol Sci.* **2020**, 7, 518-526.
11. Elerian, A.E.; Ewidea, T.A. Effect of shock wave therapy versus corticosteroid injection in management of knee osteoarthritis. *Int J Physiother.* **2016**, 3, 246-251.
12. Elgendy, M.H.; Elsamahy, S.A.; Mostafa, M.S.E.M.; Hamza, M.S.K. Efficacy of shockwave therapy versus intra-articular platelet-rich plasma injection in management of knee osteoarthritis: A randomized controlled trial. *Int J Pharm Res.* **2020**, 12, 4283-4289.
13. El-Sakka, S.S.; Hussein, M.I.; El-Barbary, A.M.; Rehan, F.S. The Effect of Shock Wave Therapy as a New Modality for Treatment of Primary Knee Osteoarthritis. *Egypt J Hosp Med.* **2019**, 75, 2092-2097.
14. Feng, Y.J.; Cao, Z. Clinical effect of Zhuifeng Tougu Capsules combined with shock wave in the treatment of knee osteoarthritis in postmenopausal women. *Chin Mod Med.* **2019**, 26, 112-114.
15. Günaydin Ö, E.; Tunay, V.B. Comparison of the added effects of kinesio taping and extracorporeal shockwave therapy to exercise alone in knee osteoarthritis. *Physiother Theory Pract.* **2020**, 1-9.
16. Guo, Y.J.; Dong, J.L.; Liu, K.; Wang, Y.J.; Mu, C.L.; Wang, P.; Shen, X.N.; Yan, L. Clinical observation on treatment of knee osteoarthritis combined with goose foot bursitis by Chinese herbal fumigation and shock wave. *China J Trad Chin Med Pharm.* **2019**, 34, 4962-4965.
17. Hammam, R.F.; Kamel, R.M.; Draz, A.H.; Azzam, A.A.; Abu El Kasem, S.T. Comparison of the effects between low- versus medium-energy radial extracorporeal shock wave therapy on knee osteoarthritis: A randomised controlled trial. *J Taibah Univ Med Sci.* **2020**, 15, 190-196.
18. He, N.; Zhang, G.J. Curative effectiveness of comprehensive rehabilitation treatment for knee osteoarthritis. *Chin J Rehabil.* **2014**, 29, 447-449.
19. He, Z.W.; Zhao, W.B. Efficacy of Arthroscopic Debridement Combined with Extracorporeal Shock Wave Treatment of Knee Osteoarthritis. *J Shenyang Med Coll.* **2016**, 18, 347-349.
20. He, Y.H.; Peng, X.; Li, Q. Clinical effect of extracorporeal shock wave combined with etoricoxib for knee osteoarthritis in the early and mid-term stage. *Shanxi Med J.* **2021**, 50, 445-446.
21. Imamura, M.; Alamino, S.; Hsing, W.T.; Alfieri, F.M.; Schmitz, C.; Battistella, L.R. Radial extracorporeal shock wave therapy for disabling pain due to severe primary knee osteoarthritis. *J Rehabil Med.* **2017**, 49, 54-62.
22. Ji, J.J.; Fang, M.Q.; Zhu, T.; Wang, Y.Y.; Chen, X.H. Radial extracorporeal shock wave acupoint therapy for elderly patients with knee osteoarthritis and analysis of its mechanism. *Chin J Mult Organ Dis Elderly.* **2021**, 20, 410-413.
23. Jiang, L.M.; Huang, S.J.; Yu, X.M.; Tao, Y.; Shen, L.H. Efficacy of extracorporeal shock wave combined joint mobilization for knee osteoarthritis. *Chin J Bone J Inj.* **2017**, 32, 1299-1301.
24. Kim, J.H.; Kim, J.Y.; Choi, C.M.; Lee, J.K.; Kee, H.S.; Jung, K.I.; Yoon, S.R. The Dose-Related Effects of Extracorporeal Shock Wave Therapy for Knee Osteoarthritis. *Ann Rehabil Med.* **2015**, 39, 616-623.
25. Kuang, G.Y.; K., Y.; Ou, L.; Chen, G.Q.; Chai, S.; Lu, M. Effects of combination of Jia Wei Duhuo Jisheng Mixture and extracorporeal shock wave therapy on knee osteoarthritis. *Chin J Gerontol.* **2017**, 37, 1708-1709.
26. Li, C.H.; Wei, Q.; Wei, S.L.; Feng, Y.G.; Wang, H.J. Clinical Observation on 167 Cases of Knee Osteoarthritis Treated with Extracorporeal Shock Wave Combining Yishen Juanbi Wan. *Rheum Arthritis.* **2018**, 7, 42-45.
27. Li, J.W.; Zheng, S.J.; Zhang, J.C.; Huang, J.J.; Liu, X.G. Effect of Acupuncture plus Different Frequency Shock-wave Interventions on Pain Reactions and Motor Function in Knee Osteoarthritis Patients. *Acupuncture Research.* **2015**, 40, 300-303.
28. Li, M.Z.; Zhang, Y.M.F.; Li, T.; Zhou, M.W. Effects of extracorporeal shock wave in the treatment for knee osteoarthritis. *Chin J Clin Healthc.* **2020**, 35, 1444-1449.
29. Li, R.X. Effect of Extracorporeal Shock Wave Therapy on Lower Limb Function and Articular Cartilage in Patients with Knee Osteoarthritis *J Clin Orthop.* **2021**, 13, 86-88.
30. Li, T.; Song, Q.Z.; Pei, J.X.; Wang, Y.L.; Niu, L.C. Observation on short term effect of extracorporeal shock wave for treating early and middle stage of knee osteoarthritis. *Chongqing Med.* **2017**, 46, 338-340.
31. Liu, B.Y.; Li, H.Y.; Xi, L.C.; Huang, S.C.; Wei, M.Z.; Wang, H.R. Comparison of early clinical outcomes of extracorporeal shock wave therapy in different energy flux densities for moderate knee osteoarthritis. *Orthop J China.* **2020**, 28, 908-912.

32. Liu, M.Y.; Li, H.; Zhang, Y.Q.; Ma, T.; Li, J.X.; Mao, Y.R.; Han, N.H. Efficacy of extracorporeal shock wave therapy in elderly patients with knee osteoarthritis and its influence on inflammatory factors. *Hainan Med J.* **2017**, 28, 4015-4017.
33. Liu, W.D.; Xue, K.L.; Tian, M. Cmparative study of pneumatic lithotripsy extracorporeal shock wave and acupotomelysis in the treatment of knee osteoarthritis. *J Cervicodynia Lumbodynia.* **2017**, 38, 64-67.
34. Liu, Y.; Wu, K.; Liu, S.T.; Zhao, Z.; Yang, J.; Xing, G.Y. Curative effect of extracorporeal shock wave therapy in elderly women with knee osteoarthritis. *Med J Chin Peop Armed Poli Forc.* **2016**, 27, 349-352.
35. Liu, Y., X.; Zhou, H.L.; Xu, L.L.; Li, T.T. Effect of extracorporeal shockwave combined with rehabilitation measures on Coll2-1 and COMP level in patients with early and middle stage knee osteoarthritis. *Orthopaedics.* **2018**, 9, 302-305.
36. Lizis, P.; Kobza, W.; Manko, G. Extracorporeal shockwave therapy vs. kinesiotherapy for osteoarthritis of the knee: A pilot randomized controlled trial. *J Back Musculoskelet Rehabil.* **2017**, 30, 1121-1128.
37. Lizis, P.; Kobza, W.; Manko, G.; Para, B. The Influence of Extracorporeal Shockwave Therapy and Kinesiotherapy on Health Status in Females with Knee Osteoarthritis: A Randomized Controlled Trial. *Int J Gen Med Surg.* **2017**, 1, 108.
38. Lizis, P.; Kobza, W.; Manko, G.; Para, B.; Jaszczur-Nowicki, J. Extracorporeal shockwave therapy is more effective than ultrasound on osteoarthritis of the knee: A pilot randomized controlled trial. *Int J Gen Med Surg.* **2018**, 2, 112.
39. Ma, G.F. The Effect of Chinese herbal fumigation combined with shock wave on treatment of knee osteoarthritis with Pes Anserine Bursitis. *Chin J Clin Ration Drug Use.* **2020**, 13, 137-139.
40. Qin, X. Clinical efficacy of extracorporeal shock wave in the treatment of osteoarthritis of the knee. *Chin Manipul Rehabil Med.* **2018**, 9, 51-52.
41. Shen, M.P.; Dai, M.C.; Qin, Q.K.; Luo, C.Y.; Pu, L.F. Effects of Extracorporeal Shock Wave Treatment on Three-Dimensional Spatiotemporal Gait Parameters in Patients with Knee Osteoarthritis. *Pract Clin Med.* **2016**, 17, 4-7.
42. Shenouda, M.M.S.S. Efficacy of extracorporeal Shock wave therapy versus mobilization with movement on pain, disability and range of motion in patients with knee osteoarthritis. *Bull Fac Phys Ther.* **2013**, 18, 65-74.
43. Shi, D.; Chen, L.; Zeng, R.H. Observation on the curative effect of silver needle combined with shock wave in the treatment of knee osteoarthritis. *Mod J Integr Trad Chin Western Med.* **2020**, 29, 1111-1114.
44. Uysal, A.; Yildizgoren, M.T.; Guler, H.; Turhanoglu, A.D. Effects of radial extracorporeal shock wave therapy on clinical variables and isokinetic performance in patients with knee osteoarthritis: a prospective, randomized, single-blind and controlled trial. *Int Orthop.* **2020**, 44, 1311-1319.
45. Wang, A.H.; Li, J.J.; Hou, G.H.; He, Y.Y. Clinical Observation of 42 Cases of Knee Osteoarthritis in Early and Middle Stage Treated with Extracorporeal Shock Wave Combined with Lower Limb Skin Traction. *Rheum Arthritis.* **2021**, 10, 15-18.
46. Wang, H.R.; Li, H.Y.; Jin, X.Y.; Liang, B.; Yin, D.; Feng, D.R.; Lv, Y.N.; Huang, Q.; Zhao, Z.X.; Liu, B.Y., et al. Clinical study on the treatment of knee osteoarthritis with extracorporeal shock wave combined with Traditional Chinese Medicine Duhuo Jisheng decoction. *Chin J New Clin Med.* **2020**, 13, 52-56.
47. Wang, L.L.; Dong, Y.; Liang, S.; Zhang, H.; Li, F.H.; Liu, F.; Tong, X.; Song, Y.P. Clinical application of radial shockwave therapy for knee osteoarthritis. *J Cervicodynia & Lumbodynia.* **2016**, 37, 354-355.
48. Wang, T.S.; Guo, P.; Li, G.; Wang, J.W. Extracorporeal Shockwave Therapy for Chronic Knee Pain: A Multicenter, Randomized Controlled Trial. *Altern Ther Health Med.* **2020**, 26, 34-37.
49. Wang, Y.Y.; Chen, X.H.; Rong, Z.; Ji, J.J.; Wu, Y.Y. Clinical observation of acupoint therapy of radio extracorporeal shock wave combined with shock wave intervention at tenderness point in the treatment of elderly patients with knee osteoarthritis. *Shanghai J Tradit Chin Med.* **2020**, 54, 98-100.
50. Wu, W.; Ye, L.; Zheng, B.J.; Feng, J.J.; Chen, X.Q.; Lui, H.R.; Xu, W.H.; Wang, X.R. Efficacy and safety of extracorporeal shock wave therapy for knee osteoarthritis. *Shanghai Med J.* **2014**, 37, 669-672.

51. Xiao, C.L.; Ding, W.T.; Tan, W.; Liu, S.S.; Yi, Z.H. Analysis of the effect of extracorporeal shock wave therapy combined with isokinetic training for knee osteoarthritis in early and mid-term stage. *Pract Clin J Integr Trad Chin Western Med.* **2021**, 21, 31-32,78.
52. Xie, B.Z.; Li, D.D.; Yin, N.; W., W.Z. A clinical study on treating knee osteoarthritis in early and middle stage by fumigation with Pingle Xitongning plus shock wave. *Clin J Chinese Med.* **2019**, 11, 109-111.
53. Xing, J.; Hu, S.H.; Tang, C.C.; Cheng, Q. Therapeutic effect of extracorporeal shock wave for patients with knee osteoarthritis. *Chin J Rehabil.* **2018**, 33, 505-507.
54. Yang, Q.X.; Zhu, W.L.; Cao, C.P.; Cao, Z.; Tan, Y.S. A clinical study on treating knee osteoarthritis by extracorporeal shock wave plus TCM medicine fumigation. *Clin J Chin Med.* **2017**, 35, 81-83.
55. Yang, Y.K.; Wu, X.H.; Yang, M.J. Clinical Observation of Shock Wave and Tendon Acupuncture Guided by Musculoskeletal Ultrasound for the Treatment of Knee Osteoarthritis. *J Pract Trad Chin Med.* **2021**, 1, 1233-1235.
56. Yu, T.C.; Chen, X.Y.; Dong, S.N.; Zhen, X.Q. Shockwave therapy for knee osteoarthritis: a single blind parallel simulation control trial. *Chin J Gerontol.* **2008**, 13, 1301-1303.
57. Yu, W.H.; Yang, W.L.; Jiao, P.Z.; Zhao, Z.J.; Feng, H.; Wen, Q.X.; Bu, Q.K.; Fan, Y.L. Clinical Study on Shock Wave Acupoint Therapy for Knee Osteoarthritis. *Hebei Med.* **2019**, 25, 1523-1526.
58. Zhang, T.; Liu, W.B.; Li, H.; Xiao, L. The Clinical Study of Radial Shock Wave Therapy in the Treatment of Knee Osteoarthritis. *Med Inn China.* **2017**, 14, 36-40.
59. Zhang, X.G.; Li, J.W.; Zheng, S.J.; Zhang, J.C.; Huang, J.J.; Liu, X.G. Observation on the Clinical Effect of Extracorporeal Shock Wave Acupuncture in the Treatment of Knee Osteoarthritis. *Guangming J Chin Med.* **2016**, 31, 2441-2444.
60. Zhang, Y.; Zhang, H. Clinical Observation of Shentongzhuyu Decoction and Extracorporeal Shock Wave Therapy to Treat Senile Knee Osteoarthritis. *Asia-Pacific Tradit Med.* **2016**, 12, 150-151.
61. Zhang, Y.F.; Li, K.X.; Wei, Q.; Feng, Y.Q. Clinical observation of extracorporeal shock wave combined with etoricoxib for early and mid-stage knee osteoarthritis *Shandong Med J.* **2020**, 60, 60-62.
62. Zhang, Y.F.; Liu, Y.; Chou, S.W.; Weng, H. Dose-related effects of radial extracorporeal shock wave therapy for knee osteoarthritis: A randomized controlled trial. *J Rehabil Med.* **2021**, 53, jrm00144.
63. Zhao, A.Q.; Xie, W.L.; Wang, Y. Effect of extracorporeal shock wave on the level of Chemerin in serum and synovia in patients with knee osteoarthritis. *Mil Med J Se China.* **2016**, 18, 498-500.
64. Zhao, Z.; Jing, R.; Shi, Z.; Zhao, B.; Ai, Q.; Xing, G. Efficacy of extracorporeal shockwave therapy for knee osteoarthritis: a randomized controlled trial. *J Surg Res.* **2013**, 185, 661-666.
65. Zhao, Z.; Shi, Z.; Yan, J.; Ai, Q.; Ding, Z.K.; Xing, G.Y. Effects of Extracorporeal Shock Wave Therapy on Early to Mid-term Knee Osteoarthritis:A Randomized Controlled Clinical Tri-al. *Chin J Rehabil Theor Pract.* **2014**, 20, 76-78.
66. Zheng, Y.L.; Yang, Q.S. The effect of extracorporeal shock wave therapy on clinical related indexes in patients with knee osteoarthritis. *Chinese J Clin.* **2021**, 49, 717-719.
67. Zhong, Z.; Liu, B.; Liu, G.; Chen, J.; Li, Y.; Chen, J.; Liu, X.; Hu, Y. A Randomized Controlled Trial on the Effects of Low-Dose Extracorporeal Shockwave Therapy in Patients With Knee Osteoarthritis. *Arch Phys Med Rehabil.* **2019**, 100, 1695-1702.
68. Zhou, S.N.; Wang, L.; Qiu, Y.Y.; Liang, L.P.; Li, J.Y.; Jin, Z. Effect of shock wave therapy combined with lower limb muscle strength exercise on patients with knee osteoarthritis pain. *Chin J Clin Healthc.* **2019**, 22, 464-467.
69. Zhu, C.Y.; Xiong, Y.S.; Liu, B.; Cheng, J.Z.; Lu, X.H. The application effect of the TCM nursing intervention on the knee osteoarthritis patients with the extracorporeal shock wave acuponit therapy. *Nurs Pract Res.* **2021**, 18, 969-973.
70. Zhu, H.M.; Yu, K.Q.; Wu, L.X.; Dai, M.Y. Clinical Study on Sinew Acupuncture Combined with Extracorporeal Shock Wave for Knee Osteoarthritis in Early and Middle Stage. *J New Chin Med.* **2021**, 53, 157-160.

Table S3. Summary of characteristics and application parameters of extracorporeal shockwave therapy.

Study (Author, year)	Energy generator	Source of energy	Device (apparatus)	Manufacturer	Shockwave treatment protocol: Application parameters (per session): Experimental group (EG): Rate (Hz)	EFD[a] (mJ/mm²)	No. of impulses	Comparator group: EFD[a] (mJ/mm²)	Treatment frequency (session/week)	Treatment duration (week)	Total treatment sessions
Aletaibieke, 2016	Radial	Pneumatic	NR	EMS, Switzerland	6	0.25	4000	0	1	4	4
Ammar 2018	Focused	Electrohydraulic	Evotron RFL0300	Swiss Tech Medical AG, Switzerland	1–4	0.05	1000	NA	1	4	4
Cai 2021	Focused	Electrohydraulic	HK.ESWO-AJ II	Wikkon, China	NR	0.202–0.205	4000	NA	2	6	12
Chang 2021	Radial	Pneumatic	MP200	Storz, Germany	12	0.13-0.25	3000 (500 impulse/acupoint)	NA	1	4	4
Chen 2014	Focused	Piezoelectric	Piezowave	Wolf, Germany	1–8	0.03–0.4	2000	NA	1	6	6
Cho 2016	Focused	Electromagnetic	Dornier Aries	DMT, Germany	NR	0.05	1000	0	1	3	3
Cui 2015	Focused	NR	NR	NR	1–2	0.15	600–1200	NA	2	4	8
Dou 2016	Focused	NR	NR	Wikkon, China	1	0.1–0.15	1000–1600	NA	1–2	3	8
Ediz 2018	Radial	Pneumatic	The ballistic system Shock Med™ of Medical Italia	Medical Italia brand (SN: EK1238092), EME, Italy	12	0.25	2500	Minimal energy pulses	2	5	10
Eftekharsadat 2020	Radial	Electromagnetic	Zimmer enPulsPro Medizin System GmbH	Zimmer, Germany	10-16	0.18	2000	NA	1–2	3	5
Elerian 2016	Radial	Pneumatic	LGT-200S	Longest, China	5	0.18	2000	Sham	1	3	3
Elgendy 2020	Radial	NR	NR	NR	5	1.25	2000	NA	1	4	4
El-Sakka 2019	Radial	Pneumatic	DolorClast	EMS, Switzerland	8	2.5-4.0	1000	NA	1	3	3
Feng 2019	Radial	Pneumatic	DolorClast Master	EMS, Switzerland	8	0.2	2000	NA	1	4	4
Gunaydin 2020	Radial	Pneumatic	DolorClast	EMS, Switzerland	6–8	0.25	2000	NA	1	6	6
Guo 2019	Radial	Pneumatic	XY-K-SONOTHERA-500	Xiangyu Medical, China	7	0.06-0.13	2000	NA	2	2	4
Hammam 2020	Radial	Pneumatic	EME S.r.l.	EME® Physio Division (Via Degli Abeti Pesaro), Italy	10	EG 1: 0.178; EG 2: 0.02	2000	0	1	4	4
He 2014	Radial	Pneumatic	BTL-5000	BTL, China	10	0.1	3000	NA	1	4	4
He 2016	Radial	Pneumatic	NR	Storz, Switzerland	9–10	0.2–0.25	2500–3000	NA	2–3	2	5
He 2021	Radial	Pneumatic	MASTERPULS MP100	STORZ MEDICAL AG, Switzerland	6	0.2	4000	NA	1	6	6
Imamura 2017	Radial	Pneumatic	DolorClast	EMS, Switzerland	8	0.1–0.16	2000	0	1	3	3
Ji 2021	Radial	Pneumatic	MP200	Storz, Switzerland	8–12	NR	3000-3500 (300 impulse/acupoint)	NA	2	12	24
Jiang 2017	Radial	Pneumatic	NR	Switzerland	6	0.25	4000	NA	1	4	4
Kim 2015	Focused	Electromagnetic	Dornier Epos Ultra	DMT, USA	NR	0.093	1000	0.04	1	3	3
Kuang 2017	Radial	Pneumatic	NR	STORZ MEDICAL AG, Switzerland	16	0.09	600-700 impulse/pain point	NA	2	5	10
Li 2018	Radial	Pneumatic	DolorClast	EMS, Switzerland	9	0.2	200	NA	1	24	24
Li 2015	Radial	Pneumatic	DolorClast	EMS, Switzerland	10–18	0.08–0.4	3000	NA	3	2	6
Li MZ 2020	Radial	Pneumatic	LGT-B2500	Longest, China	10	0.06-0.13	1000	0	2–3	4	8–12
Li RX 2021	Radial	NR	NR	NR	8	0.2	2000	NA	1	4	4
Li 2017	Radial	Pneumatic	MASTERPULS MP100	STORZ MEDICAL AG, Switzerland	6–8	0.2–0.25	4000–6000	0	1	4	4
Liu 2020	Radial	Pneumatic	MASTERPULS MP100	STORZ MEDICAL AG, Switzerland	10	EG 1: 0.22; EG 2: 0.18; EG 3: 0.14; EG 4: 0.10	2000	0.06	1	4	4
Liu MY 2017	Radial	Pneumatic	EMS	Storz, Switzerland	6–8	0.2–0.25	2000	0	1	4	4
Liu WD 2017	Radial	Pneumatic	LGT-B2500	Longest, China	6–10	0.05–0.1	2000	NA	1	6	6
Liu 2016b	Radial	Pneumatic	DolorClast	EMS, Switzerland	7	0.05–0.1	2000	NA	1	8	8

Liu YW 2018	Focused	Electromagnetic	HK ESWL-Vm	Wikkon, China	6	NR	1800–3000 (800-1000 impulse/pain point)	NA	2	3	6
Lizis 2017a	Radial	Pneumatic	Rosetta	CRT, Korea	8	0.4	1000–2000	NA	1	5	5
Lizis 2017b	Radial	Pneumatic	Rosetta	CRT, Korea	8	0.4	1000–2000	NA	1	5	5
Lizis 2018	Radial	Pneumatic	Rosetta	CRT, Korea	8	0.4	1000–2000	NA	1	5	5
Ma 2020	Radial	NR	NR	NR	7	0.06-0.13	2000	NA	5	2	10
Qin 2018	Focused	NR	NR	STORZ MEDICAL AG, Switzerland	1	0.12	2000	NA	1–2	2	3
Shen 2016	Radial	Pneumatic	DolorClast	EMS, Switzerland	10–12	0.1–0.25	1000–2500	NA	1	4	4
Shenouda 2013	Radial	NR	Shock Master	Gymna, USA	6	0.18	2000	NA	1	5	5
Shi D 2020	Focused	Electromagnetic	Dornier AR2	DMT, Germany	10	3.6-4.2	2000–4000	NA	1	4	3–4
Uysal 2020	Radial	Pneumatic	vibrolith ortho tip ESWT	ELMED, Turkey	10	0.13-0.25	2000	0.1 bar	1	3	3
Wang AH 2021	Radial	Pneumatic	NR	STORZ MEDICAL AG, Switzerland	10	0.09-0.25	2000	NA	1	4	4
Wang HR 2020	Radial	Pneumatic	MASTERPULS MP100	STORZ, Switzerland	10	0.25	2000	NA	1	4	4
Wang 2016	Radial	Pneumatic	MP100	Storz, Switzerland	12–16	0.08–0.25	8000–10000	NA	1	6	6
Wang TS 2020	Radial	Pneumatic	Sonothera	Hanil-TM Co, Korea	15	0.25	4000	0	3	10	30
Wang YY 2020	Radial	Pneumatic	MP200	Storz, Germany	12	0.13-0.25	1000-3000 (500 impulse/acupoint)	NA	1	8	8
Wu 2014	Radial	Pneumatic	MP100	Storz, Switzerland	20	0.1–0.4	3000	NA	1	4	4
Xiao 2021	Radial	Pneumatic	MASTERPULS MP100	STORZ, Switzerland	10	0.1	2000	NA	1	4	4
Xie 2019	Radial	Pneumatic	MASTERPULS MP100	STORZ, Switzerland	8	0.25	3000	NA	1	4	4
Xing 2018	Radial	Pneumatic	LGT-B2510A	Longest, China	10	0.20-0.30	2000	NA	1	4	4
Yang 2017	Radial	Pneumatic	Swiss DolorClast Smart（FT-203）	EMS, Switzerland	6	0.13-0.25	2000–4000	NA	1	4	4
Yang 2021	Radial	Pneumatic	NR	NR	7–11	0.08-0.18	2000–2500	NA	3	4	12
Yu 2008	Focused	Electrohydraulic	KDE-2001	ZJAM, China	NR	0.16–0.3	600–1000	Sham	2	1–3	2–6
Yu 2019	Radial	Pneumatic	Haobro Medical Device-V1.0		8–12	0.09-0.15	3000–3500	NA	2	9	15
Zhang 2017	Radial	Pneumatic	DolorClast	EMS, Switzerland	10–15	0.1–0.3	3000	NA	1	5	5
Zhang XG 2016	Radial	Pneumatic	Swiss DolorClast MP100	EMS, Switzerland	14	0.08-0.14	600 impulses per acupoint or pain point	NA	2–3	2	5
Zhang Y 2016	Focused	Electrohydraulic	HK.ESWO-AJ II	Wikkon, China	NR	0.16	3000	NA	3	3	8–10
Zhang 2020	Radial	Pneumatic	MASTERPULS MP100	STORZ, Switzerland	6	0.2	4000	NA	1	6	6
Zhang 2021	Radial	Pneumatic	Swiss DolorClast	EMS, Switzerland	8	EG 1: 0.24; EG 2: 0.12	2000–4000	0.02	1	4	4
Zhao 2016	Focused	NR	NR	NR	6–10	0.2–0.3	4000–6000	0	1	8	8
Zhao 2013; 2014	Radial	Pneumatic	DolorClast	EMS, Switzerland	6	0.25	4000	0	1	4	4
Zheng 2021	Radial	Pneumatic	NR	Wikkon, China	8	0.25	2000	NA	1	4	4
Zhong 2019	Radial	Pneumatic	Swiss DolorClast	EMS, Switzerland	8	0.2	1000	0.2 bar	1	4	4
Zhou 2019	Focused	Electrohydraulic	GR-TT	Beijing Glory&Health Medical, China	5–10	0.09-0.25	3000	NA	14 (2 sessions per day)	1	14
Zhu CY 2021	Radial	Pneumatic	HEMA S1	Zhuhai Hema Medical Instrument, China	6–15	0.2	3000 (300 impulse/acupoint)	NA	1	3–6	3–6
Zhu HM 2021	Radial	Pneumatic	Swiss DolorClast Smart (FT-174)	EMS, Switzerland	6–8	0.06-0.13	1000–1500	NA	5	2	10

[a]Data was reported in the unit of mJ/mm2 by the study; data reported in other units (e.g. bar, KV) was converted to mJ/mm2.

[b]TED = EFD × number of shockwave impulses.

CRT = CR Technology; EMS = Electro Medical Systems; HMT = High Medical Technology; DMT = Dornier MedTech; ZJAM = Zhongke Jian An Meditechs Company; EFD = energy flux density; TED = total energy dose (intensity × number of shockwave impulses); NR = not reported.

Table S4. Ratings of the PEDro classification scale.

Study author (year)	Overall[a]	Eligibility criteria[#]	1[b]	2[b]	3[b]	4[b]	5[b]	6[b]	7[b]	8[b]	9[b]	10[b]
Aletaibieke, 2016[¶]	8	X	X		X	X		X	X	X	X	X
Ammar 2018	5	X	X		X			X			X	X
Cai 2021	6	X	X		X				X	X	X	X
Chang 2021	6	X	X		X				X	X	X	X
Chen 2014	7	X	X	X	X				X	X	X	X
Cho 2016	8	X	X		X	X		X	X	X	X	X
Cui 2015	6	X	X		X				X	X	X	X
Dou 2016	6	X	X		X				X	X	X	X
Ediz 2018	8	X	X		X	X		X	X	X	X	X
Eftekharsadat 2020	7	X	X	X	X			X	X		X	X
Elerian 2016	9	X	X	X	X	X		X	X	X	X	X
Elgendy 2020	7	X	X		X			X	X	X	X	X
El-Sakka 2019	6	X	X		X				X	X	X	X
Feng 2019	6	X	X		X				X	X	X	X
Gunaydin 2020	6	X	X	X	X				X		X	X
Guo 2019	5	X	X		X				X		X	X
Hammam 2020[¶]	9	X	X	X	X	X		X	X	X	X	X
He 2014	6	X	X		X				X	X	X	X
He 2016	6	X	X		X				X	X	X	X
He 2021	6	X	X		X				X	X	X	X
Imamura 2017	9	X	X	X	X	X		X	X	X	X	X
Ji 2021	6	X	X		X				X	X	X	X
Jiang 2017	6	X	X		X				X	X	X	X
Kim 2015	7	X	X	X	X				X	X	X	X
Kuang 2017	6	X	X		X				X	X	X	X
Li 2018	6	X	X		X				X	X	X	X
Li 2015	6	X	X		X				X	X	X	X
Li MZ 2020[¶]	8	X	X		X	X		X	X	X	X	X
Li RX 2021	6	X	X		X				X	X	X	X
Li 2017[¶]	7	X	X		X	X		X	X		X	X
Liu 2020	6	X	X		X				X	X	X	X
Liu MY 2017[¶]	8	X	X		X	X		X	X	X	X	X
Liu WD 2017	5	X	X		X				X		X	X
Liu 2016b	6	X	X		X				X	X	X	X
Liu YW 2018	6	X	X		X				X	X	X	X
Lizis 2017a	8	X	X	X	X			X	X	X	X	X
Lizis 2017b	8	X	X	X	X			X	X	X	X	X
Lizis 2018	8	X	X	X	X			X	X	X	X	X
Ma 2020	6	X	X		X				X	X	X	X
Qin 2018	6	X	X		X				X	X	X	X
Shen 2016	6	X	X		X				X	X	X	X
Shenouda 2013	6	X	X		X				X	X	X	X
Shi D 2020	6	X	X		X				X	X	X	X
Uysal 2020	8	X	X		X	X		X	X	X	X	X
Wang AH 2021	6	X	X		X				X	X	X	X
Wang HR 2020	6	X	X		X				X	X	X	X
Wang 2016	6	X	X		X				X	X	X	X
Wang TS 2020	9	X	X		X	X	X	X	X	X	X	X
Wang YY 2020	6	X	X		X				X	X	X	X
Wu 2014	7	X	X	X	X			X	X		X	X
Xiao 2021	6	X	X		X				X	X	X	X
Xie 2019	6	X	X		X				X	X	X	X
Xing 2018	6	X	X		X				X	X	X	X
Yang 2017	7	X	X		X			X	X	X	X	X
Yang 2021	6	X	X		X				X	X	X	X
Yu 2008[¶]	7	X	X		X	X		X	X		X	X
Yu 2019	6	X	X		X				X	X	X	X
Zhang 2017	6	X	X		X				X	X	X	X
Zhang XG 2016	6	X	X		X				X	X	X	X

Zhang Y 2016	6	X	X		X				X	X	X	X
Zhang 2020	5	X	X		X				X		X	X
Zhang 2021[¶]	9	X	X	X	X	X		X	X	X	X	X
Zhao 2016[¶]	8	X	X		X	X		X	X	X	X	X
Zhao 2013; 2014	9	X	X	X	X	X		X	X	X	X	X
Zheng 2021	6	X	X		X				X	X	X	X
Zhong 2019	9	X	X		X	X	X	X	X	X	X	X
Zhou 2019	6	X	X		X				X	X	X	X
Zhu CY 2021	6	X	X		X				X	X	X	X
Zhu HM 2021	6	X	X		X				X	X	X	X
Summary*		69	69	13	69	16	2	24	68	60	69	69

PEDro = Physiotherapy Evidence Database.
[#]Not used to calculate the total score.
[¶]Score was determined by a third assessor.
*Calculated as the number of studies that satisfied the criteria.
[a]Points of methodological quality are denoted as “X” for fulfilled criteria.
[b]PEDro classification scale: 1 = random allocation, 2 = concealed allocation, 3 = similarity at the baseline, 4 = subject blinding, 5 = therapist blinding, 6 = assessor blinding, 7 = over 85% follow-up for at least one key outcome, 8 = intention-to-treat analysis, 9 = between-group statistical comparison for at least one key outcome, 10 = point and variability measures for at least one key outcome. Methodological quality: 9–10: excellent; 6–8: good; 4–5: fair; <4: poor.

Table S5. Direct pairwise comparison and network meta-analysis of mean change in pain score from baseline.

Relative effects of NMA	Direct evidence of pairwise meta-analyses																					
	V1	V2	V3	V4	V5	V6	V7	V8	V9	V10	V11	V12	V13	V14	V15	V16	V17	V18	V19	V20	V21	V22
1	MI-FoSW + CPT	.	.	.	-2.56 (-4.67, -0.45)	.	.	.	.	.	.	.	.	.	.	.	.	.	.	.	.	.
2	-1.38 (-4.70, 1.93)	MI-RaSW (Acupoint) + TCM	.	.	.	.	.	.	.	.	.	-1.62 (-3.70, 0.45)	.	.	.	.	-1.82 (-3.91, 0.27)	.	.	.	.	.
3	-2.22 (-4.76, 0.32)	-0.84 (-3.39, 1.71)	HI-RaSW + TCM	.	.	.	.	.	-1.71 (-2.91, -0.51)	.	.	.	.	.	.	.	.	.	.	.	.	-1.28 (-2.47, -0.09)
4	-2.28 (-5.54, 0.97)	-0.90 (-4.16, 2.36)	-0.06 (-2.54, 2.41)	LI-FoSW + CPT	.	.	.	.	.	.	.	.	.	.	.	.	.	.	.	.	.	-2.23 (-4.49, 0.04)
5	-2.56 (-4.67, -0.45)	-1.18 (-3.73, 1.38)	-0.34 (-1.75, 1.07)	-0.28 (-2.75, 2.19)	MI-FoSW	.	0.91 (-1.20, 3.02)	.	.	.	.	.	.	.	.	-0.55 (-2.65, 1.54)	.	.	.	.	.	-1.98 (-3.03, -0.92)
6	-2.42 (-5.57, 0.73)	-1.03 (-4.19, 2.12)	-0.20 (-2.53, 2.14)	-0.13 (-3.23, 2.96)	0.14 (-2.19, 2.48)	MI-RaSW + TCM	.	.	.	.	.	.	.	.	.	.	.	.	.	.	.	-2.09 (-4.20, 0.02)
7	-2.58 (-5.42, 0.26)	-1.19 (-4.21, 1.83)	-0.36 (-2.50, 1.79)	-0.29 (-3.25, 2.66)	-0.01 (-1.91, 1.88)	-0.16 (-2.99, 2.68)	MI-FoSW + TCM	.	.	.	.	.	.	.	.	.	.	.	.	.	.	-1.01 (-3.12, 1.10)
8	-2.77 (-5.29, -0.25)	-1.39 (-3.91, 1.14)	-0.55 (-1.91, 0.82)	-0.49 (-2.94, 1.97)	-0.21 (-1.59, 1.18)	-0.35 (-2.67, 1.96)	-0.19 (-2.32, 1.93)	HI-RaSW + CPT	-1.16 (-3.27, 0.96)	.	.	.	.	.	.	.	.	.	.	.	-0.03 (-2.11, 2.05)	-2.20 (-3.30, -1.09)
9	-2.87 (-5.27, -0.47)	-1.49 (-3.89, 0.92)	-0.65 (-1.65, 0.35)	-0.59 (-2.91, 1.74)	-0.31 (-1.45, 0.83)	-0.45 (-2.63, 1.73)	-0.29 (-2.27, 1.68)	-0.10 (-1.15, 0.95)	HI-RaSW	.	-0.48 (-1.95, 1.00)	.	.	.	.	.	.	.	.	.	-2.89 (-4.41, -1.38)	-1.68 (-2.29, -1.07)
10	-2.85 (-6.00, 0.30)	-1.46 (-4.62, 1.70)	-0.62 (-2.96, 1.71)	-0.56 (-3.66, 2.54)	-0.28 (-2.62, 2.06)	-0.43 (-3.41, 2.56)	-0.27 (-3.11, 2.57)	-0.08 (-2.40, 2.25)	0.03 (-2.16, 2.21)	HI-FoSW + TCM	.	.	.	.	.	.	.	.	.	.	.	-1.67 (-3.78, 0.45)
11	-2.90 (-5.36, -0.45)	-1.52 (-3.92, 0.88)	-0.68 (-1.90, 0.54)	-0.62 (-3.00, 1.76)	-0.34 (-1.59, 0.91)	-0.48 (-2.72, 1.75)	-0.33 (-2.36, 1.71)	-0.13 (-1.32, 1.06)	-0.03 (-0.89, 0.82)	-0.06 (-2.30, 2.18)	MI-RaSW	.	.	.	.	.	0.36 (-1.73, 2.44)	.	.	.	-2.43 (-3.93, -0.94)	-1.64 (-2.60, -0.69)
12	-3.01 (-6.92, 0.90)	-1.62 (-3.70, 0.45)	-0.79 (-4.07, 2.50)	-0.72 (-4.59, 3.14)	-0.45 (-3.74, 2.85)	-0.59 (-4.37, 3.19)	-0.43 (-4.10, 3.23)	-0.24 (-3.51, 3.03)	-0.14 (-3.31, 3.03)	-0.16 (-3.95, 3.62)	-0.11 (-3.28, 3.07)	MI-RaSW (Acupoint) + CPT	.	.	.	.	.	.	.	.	.	.
13	-3.12 (-6.25, 0.01)	-1.73 (-4.87, 1.40)	-0.90 (-3.20, 1.41)	-0.84 (-3.91, 2.24)	-0.56 (-2.86, 1.75)	-0.70 (-3.66, 2.26)	-0.54 (-3.36, 2.27)	-0.35 (-2.64, 1.94)	-0.25 (-2.40, 1.90)	-0.27 (-3.24, 2.69)	-0.22 (-2.43, 1.99)	-0.11 (-3.87, 3.65)	HI-FoSW + CPT	.	.	.	.	.	.	.	.	-1.39 (-3.47, 0.69)
14	-3.14 (-6.27, -0.01)	-1.76 (-4.90, 1.38)	-0.92 (-3.23, 1.39)	-0.86 (-3.94, 2.22)	-0.58 (-2.89, 1.73)	-0.72 (-3.69, 2.24)	-0.57 (-3.38, 2.25)	-0.37 (-2.67, 1.92)	-0.27 (-2.43, 1.88)	-0.30 (-3.27, 2.67)	-0.24 (-2.46, 1.98)	-0.13 (-3.90, 3.63)	-0.02 (-2.97, 2.92)	LI-FoSW + TCM	.	.	.	.	.	.	.	-1.37 (-3.46, 0.72)
15	-3.14 (-5.81, -0.46)	-1.75 (-4.40, 0.90)	-0.91 (-2.55, 0.72)	-0.85 (-3.47, 1.76)	-0.57 (-2.22, 1.08)	-0.72 (-3.20, 1.77)	-0.56 (-2.86, 1.75)	-0.37 (-1.94, 1.21)	-0.26 (-1.66, 1.13)	-0.29 (-2.78, 2.20)	-0.23 (-1.69, 1.23)	-0.13 (-3.49, 3.24)	-0.02 (-2.47, 2.44)	0.01 (-2.46, 2.47)	LI-RaSW + TCM	.	.	.	.	.	-1.42 (-2.91, 0.07)	-1.21 (-3.30, 0.88)
16	-3.23 (-5.88, -0.57)	-1.84 (-4.70, 1.02)	-1.00 (-2.91, 0.91)	-0.94 (-3.73, 1.85)	-0.66 (-2.27, 0.94)	-0.81 (-3.47, 1.86)	-0.65 (-3.04, 1.74)	-0.46 (-2.35, 1.43)	-0.36 (-2.07, 1.36)	-0.38 (-3.05, 2.29)	-0.32 (-2.12, 1.47)	-0.22 (-3.75, 3.32)	-0.11 (-2.75, 2.53)	-0.08 (-2.73, 2.56)	-0.09 (-2.18, 2.00)	LI-FoSW	.	.	.	.	.	-1.16 (-3.42, 1.11)
17	-3.20 (-5.78, -0.63)	-1.82 (-3.91, 0.27)	-0.98 (-2.44, 0.48)	-0.92 (-3.43, 1.59)	-0.64 (-2.11, 0.83)	-0.79 (-3.15, 1.58)	-0.63 (-2.81, 1.55)	-0.43 (-1.85, 0.98)	-0.33 (-1.52, 0.85)	-0.36 (-2.73, 2.01)	-0.30 (-1.48, 0.88)	-0.20 (-3.14, 2.75)	-0.09 (-2.43, 2.26)	-0.06 (-2.41, 2.29)	-0.07 (-1.70, 1.56)	0.02 (-1.93, 1.98)	MI-RaSW (Acupoint)	.	.	.	-1.40 (-3.49, 0.69)	-0.92 (-2.38, 0.55)
18	-3.31 (-5.83, -0.79)	-1.92 (-4.46, 0.61)	-1.08 (-2.46, 0.29)	-1.02 (-3.47, 1.43)	-0.74 (-2.12, 0.63)	-0.89 (-3.20, 1.42)	-0.73 (-2.85, 1.39)	-0.54 (-1.88, 0.81)	-0.44 (-1.53, 0.65)	-0.46 (-2.78, 1.85)	-0.40 (-1.61, 0.80)	-0.30 (-3.57, 2.98)	-0.19 (-2.47, 2.10)	-0.16 (-2.45, 2.13)	-0.17 (-1.79, 1.44)	-0.08 (-1.96, 1.80)	-0.10 (-1.53, 1.33)	HI-FoSW	.	.	.	-1.21 (-2.15, -0.26)
19	-3.33 (-5.79, -0.88)	-1.95 (-4.40, 0.51)	-1.11 (-2.36, 0.14)	-1.05 (-3.44, 1.34)	-0.77 (-2.03, 0.49)	-0.91 (-3.16, 1.33)	-0.76 (-2.80, 1.29)	-0.56 (-1.76, 0.64)	-0.46 (-1.39, 0.46)	-0.49 (-2.74, 1.76)	-0.43 (-1.47, 0.61)	-0.32 (-3.54, 2.89)	-0.21 (-2.43, 2.00)	-0.19 (-2.41, 2.03)	-0.20 (-1.65, 1.26)	-0.11 (-1.91, 1.69)	-0.13 (-1.42, 1.16)	-0.03 (-1.24, 1.19)	MI-RaSW + CPT	-1.59 (-3.77, 0.59)	-1.51 (-2.99, -0.03)	-1.01 (-1.88, -0.15)
20	-3.55 (-6.28, -0.81)	-2.16 (-4.91, 0.59)	-1.32 (-3.06, 0.42)	-1.26 (-3.94, 1.41)	-0.98 (-2.73, 0.76)	-1.13 (-3.67, 1.42)	-0.97 (-3.34, 1.41)	-0.78 (-2.49, 0.94)	-0.68 (-2.20, 0.85)	-0.70 (-3.25, 1.85)	-0.64 (-2.25, 0.97)	-0.54 (-3.98, 2.91)	-0.43 (-2.95, 2.10)	-0.40 (-2.93, 2.13)	-0.41 (-2.34, 1.51)	-0.32 (-2.49, 1.85)	-0.34 (-2.12, 1.44)	-0.24 (-1.95, 1.47)	-0.21 (-1.73, 1.30)	LI-RaSW + CPT	.	-1.53 (-3.06, 0.01)
21	-4.47 (-6.93, -2.02)	-3.09 (-5.49, -0.69)	-2.25 (-3.47, -1.02)	-2.19 (-4.57, 0.20)	-1.91 (-3.16, -0.66)	-2.05 (-4.29, 0.18)	-1.89 (-3.93, 0.15)	-1.70 (-2.81, -0.59)	-1.60 (-2.46, -0.74)	-1.63 (-3.87, 0.62)	-1.57 (-2.51, -0.62)	-1.46 (-4.63, 1.71)	-1.35 (-3.56, 0.86)	-1.33 (-3.55, 0.89)	-1.34 (-2.58, -0.10)	-1.25 (-3.04, 0.55)	-1.27 (-2.45, -0.09)	-1.17 (-2.37, 0.04)	-1.14 (-2.07, -0.21)	-0.93 (-2.51, 0.66)	LI-RaSW	-1.09 (-2.57, 0.40)
22	-4.51 (-6.85, -2.18)	-3.13 (-5.48, -0.78)	-2.29 (-3.29, -1.29)	-2.23 (-4.49, 0.04)	-1.95 (-2.95, -0.95)	-2.09 (-4.20, 0.02)	-1.93 (-3.83, -0.04)	-1.74 (-2.70, -0.78)	-1.64 (-2.19, -1.10)	-1.67 (-3.78, 0.45)	-1.61 (-2.36, -0.86)	-1.50 (-4.64, 1.63)	-1.39 (-3.47, 0.69)	-1.37 (-3.46, 0.72)	-1.38 (-2.69, -0.07)	-1.29 (-2.91, 0.34)	-1.31 (-2.39, -0.23)	-1.21 (-2.15, -0.26)	-1.18 (-1.94, -0.41)	-0.97 (-2.39, 0.46)	-0.04 (-0.79, 0.71)	UC

Pairwise (upper right portion) and network (lower left portion) meta-analysis results are presented for mean change (from baseline) in pain outcomes. Interventions are reported in order of efficacy ranked according to P scores. Effect estimation is presented in standardized mean difference (SMD) with 95% CI. Significant results are marked in red.

NMA, network meta-analysis; FoSW, focused shock wave; RaSW, radial shock wave; HI, high energy; MI, medium energy; LI, low energy; CPT, conventional physical therapy; TCM, traditional Chinese medicine; UC, usual care.

Table S6. Direct pairwise comparison and network meta-analysis of mean change in global function from baseline.

Relative effects of NMA	Direct evidence of pairwise meta-analyses																				
	V1	V2	V3	V4	V5	V6	V7	V8	V9	V10	V11	V12	V13	V14	V15	V16	V17	V18	V19	V20	V21
1	MI-RaSW (Acupoint) + TCM	0.85 (-1.42, 3.12)	.	2.33 (0.03, 4.63)	.	.	.	.	.	.	.	.	.	.	.	.	.	.	.	.	.
2	0.85 (-1.42, 3.12)	MI-RaSW (Acupoint) + CPT	.	.	.	.	.	.	.	.	.	.	.	.	.	.	.	.	.	.	.
3	2.29 (-0.53, 5.12)	1.45 (-2.18, 5.07)	HI-RaSW + TCM	.	.	.	.	.	.	.	1.44 (0.12, 2.75)	.	.	.	.	.	.	.	.	.	1.88 (0.57, 3.19)
4	2.33 (0.03, 4.63)	1.49 (-1.75, 4.72)	0.04 (-1.60, 1.68)	MI-RaSW (Acupoint)	1.29 (-0.32, 2.90)	.	.	.	.	.	.	.	.	.	.	.	.	1.54 (-0.75, 3.84)	.	.	1.01 (-1.27, 3.28)
5	2.69 (0.09, 5.29)	1.84 (-1.61, 5.29)	0.39 (-0.95, 1.74)	0.35 (-0.86, 1.57)	MI-RaSW	.	.	.	.	.	0.12 (-1.49, 1.73)	.	.	.	.	.	.	1.11 (-0.52, 2.74)	.	.	2.79 (1.72, 3.86)
6	2.50 (-1.17, 6.18)	1.66 (-2.66, 5.98)	0.21 (-2.59, 3.01)	0.17 (-2.69, 3.04)	-0.18 (-2.89, 2.53)	MI-FoSW + CPT	.	.	.	.	.	0.85 (-1.43, 3.13)	.	.	.	.	.	.	.	.	.
7	2.82 (-0.65, 6.30)	1.98 (-2.18, 6.13)	0.53 (-2.01, 3.06)	0.49 (-2.12, 3.10)	0.14 (-2.30, 2.57)	0.32 (-3.13, 3.77)	HI-FoSW + CPT	.	.	.	.	.	.	.	.	.	.	.	.	.	1.97 (-0.32, 4.26)
8	3.08 (0.29, 5.88)	2.24 (-1.36, 5.84)	0.79 (-0.70, 2.28)	0.75 (-0.84, 2.34)	0.40 (-0.90, 1.69)	0.58 (-2.20, 3.36)	0.26 (-2.25, 2.77)	HI-RaSW + CPT	.	.	3.32 (0.96, 5.69)	.	.	.	.	.	.	0.69 (-1.60, 2.98)	.	.	1.61 (0.43, 2.80)
9	3.16 (0.45, 5.87)	2.31 (-1.22, 5.85)	0.87 (-0.47, 2.20)	0.83 (-0.61, 2.26)	0.47 (-0.63, 1.58)	0.66 (-2.04, 3.35)	0.34 (-2.08, 2.76)	0.08 (-1.20, 1.35)	MI-RaSW + CPT	.	.	.	.	.	.	.	1.46 (-0.91, 3.83)	1.94 (0.31, 3.57)	-0.95 (-3.23, 1.33)	.	1.61 (0.73, 2.49)
10	3.16 (0.05, 6.27)	2.31 (-1.54, 6.17)	0.87 (-1.25, 2.98)	0.83 (-1.27, 2.92)	0.47 (-1.45, 2.40)	0.66 (-2.51, 3.82)	0.34 (-2.60, 3.27)	0.08 (-1.96, 2.11)	-0.00 (-1.92, 1.91)	LI-RaSW + TCM	.	.	.	.	.	.	.	1.13 (-0.50, 2.75)	.	.	.
11	3.25 (0.59, 5.91)	2.41 (-1.09, 5.90)	0.96 (-0.13, 2.05)	0.92 (-0.42, 2.26)	0.57 (-0.37, 1.50)	0.75 (-1.89, 3.39)	0.43 (-1.93, 2.79)	0.17 (-0.97, 1.31)	0.09 (-0.87, 1.05)	0.09 (-1.79, 1.98)	HI-RaSW	.	.	.	.	.	.	0.98 (-0.66, 2.61)	.	.	1.65 (1.01, 2.29)
12	3.35 (0.47, 6.23)	2.51 (-1.16, 6.17)	1.06 (-0.56, 2.68)	1.02 (-0.71, 2.75)	0.67 (-0.79, 2.12)	0.85 (-1.43, 3.13)	0.53 (-2.06, 3.11)	0.27 (-1.32, 1.86)	0.19 (-1.24, 1.63)	0.19 (-2.00, 2.39)	0.10 (-1.23, 1.43)	MI-FoSW	.	-1.04 (-3.35, 1.27)	.	.	.	.	.	1.39 (-0.91, 3.70)	1.56 (0.23, 2.88)
13	3.38 (0.56, 6.19)	2.53 (-1.08, 6.15)	1.08 (-0.42, 2.59)	1.05 (-0.58, 2.67)	0.69 (-0.63, 2.02)	0.87 (-1.90, 3.65)	0.56 (-1.96, 3.07)	0.29 (-1.17, 1.76)	0.22 (-1.08, 1.52)	0.22 (-1.89, 2.33)	0.13 (-1.06, 1.31)	0.03 (-1.56, 1.61)	HI-FoSW	.	.	.	.	.	.	.	1.41 (0.38, 2.45)
14	3.49 (0.58, 6.41)	2.65 (-1.05, 6.34)	1.20 (-0.48, 2.88)	1.16 (-0.63, 2.95)	0.81 (-0.72, 2.33)	0.99 (-1.79, 3.77)	0.67 (-1.95, 3.29)	0.41 (-1.24, 2.06)	0.33 (-1.17, 1.84)	0.33 (-1.90, 2.57)	0.24 (-1.16, 1.65)	0.14 (-1.44, 1.73)	0.12 (-1.53, 1.76)	MI-FoSW + TCM	.	.	.	.	.	.	1.03 (-0.30, 2.35)
15	3.86 (0.32, 7.40)	3.01 (-1.19, 7.22)	1.57 (-1.06, 4.19)	1.53 (-1.16, 4.22)	1.17 (-1.35, 3.70)	1.36 (-2.16, 4.87)	1.04 (-2.27, 4.34)	0.78 (-1.82, 3.38)	0.70 (-1.81, 3.21)	0.70 (-2.31, 3.71)	0.61 (-1.85, 3.06)	0.51 (-2.16, 3.18)	0.48 (-2.12, 3.08)	0.37 (-2.34, 3.07)	LI-FoSW + CPT	.	.	.	.	.	0.93 (-1.45, 3.32)
16	4.04 (0.56, 7.53)	3.20 (-0.96, 7.36)	1.75 (-0.79, 4.29)	1.71 (-0.90, 4.33)	1.36 (-1.08, 3.80)	1.54 (-1.91, 4.99)	1.22 (-2.02, 4.46)	0.96 (-1.56, 3.48)	0.88 (-1.54, 3.31)	0.89 (-2.06, 3.83)	0.79 (-1.58, 3.16)	0.69 (-1.90, 3.29)	0.67 (-1.85, 3.19)	0.55 (-2.08, 3.18)	0.18 (-3.13, 3.50)	HI-FoSW + TCM	.	.	.	.	0.75 (-1.55, 3.05)
17	3.95 (0.91, 6.99)	3.10 (-0.69, 6.89)	1.65 (-0.24, 3.55)	1.62 (-0.37, 3.60)	1.26 (-0.49, 3.01)	1.45 (-1.56, 4.45)	1.13 (-1.64, 3.89)	0.87 (-1.00, 2.73)	0.79 (-0.84, 2.42)	0.79 (-1.60, 3.17)	0.70 (-0.96, 2.35)	0.60 (-1.36, 2.56)	0.57 (-1.29, 2.43)	0.45 (-1.56, 2.47)	0.09 (-2.76, 2.93)	-0.10 (-2.87, 2.68)	LI-RaSW + CPT	.	.	.	1.22 (-0.45, 2.89)
18	4.29 (1.63, 6.94)	3.44 (-0.05, 6.93)	1.99 (0.63, 3.35)	1.96 (0.63, 3.28)	1.60 (0.56, 2.64)	1.78 (-0.93, 4.50)	1.46 (-0.98, 3.91)	1.20 (-0.02, 2.43)	1.13 (0.11, 2.14)	1.13 (-0.50, 2.75)	1.03 (0.08, 1.99)	0.94 (-0.54, 2.41)	0.91 (-0.44, 2.25)	0.79 (-0.75, 2.34)	0.43 (-2.11, 2.96)	0.24 (-2.21, 2.70)	0.34 (-1.41, 2.09)	LI-RaSW	.	.	0.49 (-1.15, 2.12)
19	4.66 (1.64, 7.69)	3.82 (0.04, 7.60)	2.37 (0.50, 4.24)	2.33 (0.37, 4.29)	1.98 (0.25, 3.71)	2.16 (-0.83, 5.15)	1.84 (-0.91, 4.59)	1.58 (-0.26, 3.42)	1.50 (-0.10, 3.11)	1.50 (-0.86, 3.87)	1.41 (-0.22, 3.04)	1.31 (-0.63, 3.25)	1.29 (-0.56, 3.13)	1.17 (-0.82, 3.16)	0.80 (-2.03, 3.63)	0.62 (-2.14, 3.38)	0.72 (-1.43, 2.87)	0.38 (-1.35, 2.10)	MI-RaSW + TCM	.	-0.43 (-2.06, 1.20)
20	4.74 (1.57, 7.91)	3.90 (0.00, 7.79)	2.45 (0.36, 4.54)	2.41 (0.23, 4.59)	2.06 (0.09, 4.02)	2.24 (-0.65, 5.12)	1.92 (-0.98, 4.82)	1.66 (-0.41, 3.72)	1.58 (-0.37, 3.53)	1.58 (-0.98, 4.14)	1.49 (-0.39, 3.37)	1.39 (-0.38, 3.16)	1.36 (-0.70, 3.43)	1.25 (-0.88, 3.38)	0.88 (-2.10, 3.86)	0.70 (-2.21, 3.61)	0.79 (-1.57, 3.16)	0.45 (-1.53, 2.44)	0.08 (-2.27, 2.42)	LI-FoSW	0.06 (-2.37, 2.48)
21	4.79 (2.17, 7.41)	3.95 (0.48, 7.41)	2.50 (1.41, 3.59)	2.46 (1.21, 3.71)	2.11 (1.28, 2.93)	2.29 (-0.29, 4.87)	1.97 (-0.32, 4.26)	1.71 (0.67, 2.75)	1.63 (0.85, 2.42)	1.63 (-0.20, 3.47)	1.54 (0.96, 2.12)	1.44 (0.24, 2.64)	1.41 (0.38, 2.45)	1.30 (0.02, 2.58)	0.93 (-1.45, 3.32)	0.75 (-1.55, 3.05)	0.84 (-0.71, 2.39)	0.51 (-0.35, 1.37)	0.13 (-1.39, 1.65)	0.05 (-1.73, 1.84)	UC

Pairwise (upper right portion) and network (lower left portion) meta-analysis results are presented for mean change (from baseline) in pain outcomes. Interventions are reported in order of efficacy ranked according to P scores. Effect estimation is presented in standardized mean difference (SMD) with 95% CI. Significant results are marked in red.

NMA, network meta-analysis; FoSW, focused shock wave; RaSW, radial shock wave; HI, high energy; MI, medium energy; LI, low energy; CPT, conventional physical therapy; TCM, traditional Chinese medicine; UC, usual care.

Table S7. Direct pairwise comparison and network meta-analysis for disease activity.

Relative effects of NMA	Direct evidence of pairwise meta-analyses								
	V1	V2	V3	V4	V5	V6	V7	V8	V9
1	HI-FoSW	.	.	.	.	.	.	.	-5.21 (-7.43, -3.00)
2	-3.00 (-7.62, 1.61)	MI-RaSW (Acupoint) + TCM	.	.	.	.	.	-1.47 (-4.34, 1.40)	.
3	-3.82 (-8.43, 0.79)	-0.82 (-6.54, 4.90)	MI-RaSW + TCM	-0.45 (-3.32, 2.41)	.	.	.	.	.
4	-4.28 (-7.89, -0.67)	-1.27 (-6.23, 3.68)	-0.45 (-3.32, 2.41)	MI-RaSW	.	.	.	.	-0.94 (-3.79, 1.92)
5	-4.40 (-7.81, -0.99)	-1.40 (-6.21, 3.41)	-0.58 (-5.39, 4.23)	-0.13 (-3.99, 3.73)	HI-RaSW + TCM	.	-0.47 (-3.31, 2.37)	.	-0.48 (-3.32, 2.36)
6	-4.43 (-8.04, -0.82)	-1.43 (-6.38, 3.53)	-0.61 (-5.56, 4.35)	-0.15 (-4.19, 3.89)	-0.03 (-3.89, 3.83)	MI-RaSW + CPT	.	.	-0.78 (-3.64, 2.07)
7	-4.54 (-7.30, -1.78)	-1.54 (-5.91, 2.83)	-0.72 (-5.09, 3.65)	-0.27 (-3.56, 3.03)	-0.14 (-2.74, 2.46)	-0.11 (-3.41, 3.19)	HI-RaSW	.	-0.67 (-2.32, 0.98)
8	-4.47 (-8.09, -0.86)	-1.47 (-4.34, 1.40)	-0.65 (-5.60, 4.30)	-0.20 (-4.23, 3.84)	-0.07 (-3.93, 3.79)	-0.04 (-4.08, 4.00)	0.07 (-3.23, 3.37)	MI-RaSW (Acupoint)	-0.74 (-3.60, 2.12)
9	-5.21 (-7.43, -3.00)	-2.21 (-6.26, 1.84)	-1.39 (-5.43, 2.66)	-0.94 (-3.79, 1.92)	-0.81 (-3.41, 1.79)	-0.78 (-3.64, 2.07)	-0.67 (-2.32, 0.98)	-0.74 (-3.60, 2.12)	UC

Pairwise (upper right portion) and network (lower left portion) meta-analysis results are presented for mean change (from baseline) in pain outcomes. Interventions are reported in order of efficacy ranked according to P scores. Effect estimation is presented in standardized mean difference (SMD) with 95% CI. Significant results are marked in red.

NMA, network meta-analysis; FoSW, focused shock wave; RaSW, radial shock wave; HI, high energy; MI, medium energy; CPT, conventional physical therapy; TCM, traditional Chinese medicine; UC, usual care.

Table S8. Associations of moderators with treatment efficiency for all outcome measures.

	Knee pain[a]						Global function[a]						Disease activity[a]					
Moderator	N	B	SE	Median	95% CI		N	B	SE	Median	95% CI		N	B	SE	Median	95% CI	
Participant factor																		
Age	61	0.643	0.0024	0.641	−0.296,	1.591	62	0.031	0.0101	0.033	−1.287,	1.301	11	0.826	0.0641	0.937	−6.993,	9.051
BMI	27	−0.502	0.0037	−0.507	−1.937,	0.963	28	−0.061	0.0096	−0.047	−1.273,	1.079	4	NA				
Sex distribution[b]	58	0.051	0.0060	0.056	−0.708,	0.781	60	−0.252	0.0084	−0.234	−1.323,	0.805	11	0.107	0.0720	0.510	−10.205,	8.734
Area of population[c]	62	0.865	0.0065	0.860	0.070,	1.709	63	−0.294	0.0073	−0.291	−1.211,	0.640	11	1.633	0.0028	0.158	−0.443,	0.810
Disease duration	47	−0.091	0.0065	−0.087	−0.915,	0.699	48	0.406	0.0096	0.396	−0.829,	1.630	11	5.195	0.194	2.786	−12.536,	28.315
Study design factor																		
PEDro score	62	−0.883	0.0020	−0.881	−1.681,	−0.105	63	0.849	0.0083	0.846	−0.183,	1.893	11	−2.322	0.0513	−2.442	−8.588,	6.299
Follow-up duration	62	−0.327	0.0153	−0.329	−1.674,	0.999	63	0.338	0.0104	0.339	−0.960,	1.638	11	−6.979	0.2651	0.943	−45.417,	7.100
Intervention factor																		
Wave[d]	62	4.372	0.1310	4.561	−4.712,	13.468	63	−1.102	0.1201	−0.164	−11.421,	6.554	11	16.335	0.2938	18.528	−14.651,	47.504
EFD	61	−0.550	0.0155	−0.563	−1.619,	0.610	63	0.548	0.0256	0.512	−1.144,	2.517	10	−6.114	0.0971	−3.547	−17.113,	5.703
Treatment composition[e]	62	0.492	0.0168	0.520	−0.858,	1.697	63	−1.481	0.0210	−1.488	−2.891,	0.030	11	−12.170	0.1005	−7.428	−18.700,	4.214
Treatment duration	62	−0.190	0.0087	−0.174	−0.981,	0.534	63	0.246	0.0083	0.229	−0.778,	1.273	11	−7.193	0.5780	−13.500	−61.064,	41.787

[a]Data represents the change in effects associated with the moderator indicated. B, beta coefficient; SE, standard error; 95% CI, 95% credibility interval.

[b]The proportion of female participants in sample

[c]Code for regression model: Africa = 1; Asian = 2; Europe = 3.

[d]Code for regression model: focused shock wave = 1; radial shock wave = 2.

[e]Code for regression model: monotherapy= 1; combined treatment = 2.

BMI, body mass index; PEDro, Physiotherapy Evidence Database; EFD, energy flux density.

Table S9. Compliance and adverse events reported by the included trials.

Study	ESWT group						Comparator group		
	ESWT alone		ESWT + CPT		ESWT + TCM		Placebo or Non-ESWT	CPT	TCM
	Related to ESWT	Unrelated to ESWT	Related to ESWT	Unrelated to ESWT	Related to ESWT	Unrelated to ESWT			
Aletaibieke, 2016	NR	NR					NR		
Ammar 2018			X	None				X	
Cai 2021	None	X					X		
Chang 2021	None	X			None	X			
Chen 2014			X	None			X	X	
Cho 2016	None	None					None		
Cui 2015	None	None			None	None			None
Dou 2016	NR	NR						NR	
Ediz 2018			None	None			None	None	
Eftekharsadat 2020			NR	NR				NR	
Elerian 2016	NR	NR					NR		
Elgendy 2020			NR	NR				NR	
El-Sakka 2019			NR	NR				NR	
Feng 2019	NR	NR			NR	NR			
Gunaydin 2020			NR	NR				NR	
Guo 2019					NR	NR			NR
Hammam 2020			NR	NR			NR	NR	
He 2014			NR	NR				NR	
He 2016	NR	NR					NR		
He 2021	NR	NR					NR		
Imamura 2017	None	None					None		
Ji 2021	X	None					X		
Jiang 2017	NR	NR	NR	NR				NR	
Kim 2015	X	None					None		
Kuang 2017	X	None			None	X			
Li 2018					None	X	X		
Li 2015	NR	NR							NR
Li MZ 2020			NR	NR			NR	NR	
Li RX 2021	NR	NR						NR	
Li 2017	X	None					X		
Liu 2020	X	None					None		
Liu MY 2017	NR	NR					NR		
Liu WD 2017	NR	NR							NR
Liu 2016b	None	None					None		
Liu YW 2018					NR	NR	NR		
Lizis 2017a	None	None						None	
Lizis 2017b	None	None						None	
Lizis 2018	None	None						None	
Ma 2020					NR	NR			NR
Qin 2018	NR	NR						NR	
Shen 2016	NR							NR	NR
Shenouda 2013			NR	NR				NR	
Shi D 2020					NR	NR	NR		
Uysal 2020			NR	NR			NR	NR	
Wang AH 2021			NR	NR				NR	
Wang HR 2020	NR	NR			NR	NR			
Wang 2016	NR	NR						NR	
Wang TS 2020	NR	NR					NR		
Wang YY 2020	NR	NR							
Wu 2014	X	None					X		
Xiao 2021			NR	NR				NR	
Xie 2019	NR	NR			NR	NR			NR
Xing 2018	NR	NR					NR		

To be continued.

Table S9. Continued.

Study	ESWT group						Comparator group		
	ESWT alone		ESWT + CPT		ESWT + TCM		Placebo or Non-ESWT	CPT	TCM
	Related to ESWT	Unrelated to ESWT	Related to ESWT	Unrelated to ESWT	Related to ESWT	Unrelated to ESWT			
Yang 2017			NR	NR	NR	NR		NR	NR
Yang 2021					None	None		None	None
Yu 2008	NR	NR					NR		
Yu 2019	NR	NR							
Zhang 2017			X	None				X	
Zhang XG 2016	NR	NR							
Zhang Y 2016					NR	NR	NR		
Zhang 2020	NR	NR					NR		
Zhang 2021	None	None					None		
Zhao 2016	NR	NR					NR		
Zhao 2013; 2014	None	None					X		
Zheng 2021	NR	NR					NR		
Zhong 2019			X	X				X	
Zhou 2019	NR	NR	NR	NR					
Zhu CY 2021			NR	NR	NR	NR			
Zhu HM 2021	None	X			None	X			

[a]The occurrence of adverse events is denoted as "X"; NR = data of the complications or adverse events were not reported.
